# Density and Viscosity of Binary Mixtures of Thiocyanate Ionic Liquids + Water as a Function of Temperature

**DOI:** 10.1007/s10953-012-9875-7

**Published:** 2012-08-21

**Authors:** U. Domańska, M. Królikowska

**Affiliations:** 1 Department of Physical Chemistry, Faculty of Chemistry, Warsaw University of Technology, Noakowskiego 3, 00-664 Warsaw, Poland; 2 Thermodynamic Research Unit, School of Chemical Engineering, University of KwaZulu-Natal, Howard College Campus, King George V Avenue, Durban, 4001 South Africa

**Keywords:** Experimental density, Viscosity, Excess molar volume, Deviation in viscosity, Molecular interactions, Ionic liquid, [BMIM][SCN], [BMPy][SCN], [BMPYR][SCN], [BMPIP][SCN]

## Abstract

Densities and viscosities have been determined for binary mixtures of the ionic liquids (ILs) 1-butyl-3-methylimidazolium thiocyanate [BMIM][SCN], or 1-butyl-4-methylpyridinium thiocyanate [BMPy][SCN], or 1-butyl-1-methylpyrrolidinium thiocyanate [BMPYR][SCN], or 1-butyl-1-methylpiperidinium thiocyanate [BMPIP][SCN] with water over wide range of temperatures (298.15–348.15) K and ambient pressure. The thermal properties of [BMPy][SCN], i.e. glass transition temperature and the heat capacity at glass transition, have been measured using a differential scanning microcalorimetry, DSC. The decomposition of [BMPy][SCN] was detected. The density and viscosity correlations for these systems have been made using an empirical second-order polynomial and by the Vogel–Fulcher–Tammann equation, respectively. The concentration dependences have been described by polynomials. The excess molar volumes and deviations in viscosity have been calculated from the experimental values and were correlated by Redlich–Kister polynomial expansions. The variations of these parameters, with compositions of the mixtures and temperature, have been discussed in terms of molecular interactions. A qualitative analysis of the trend of properties with composition and temperature was performed. Further, the excess partial molar volumes, $V_{1}^{\mathrm{E}}$ and $V_{2}^{\mathrm{E}}$, were calculated and discussed. The isobaric expansivities (coefficient of thermal expansion), *α*, and the excess isobaric expansivities, *α* ^E^, were determined for four ILs and their mixtures with water. The results indicate that the interactions of thiocyanate ILs with water is not as strong as with alcohols, which is shown by the positive/slightly negative excess molar volumes in these binary systems.

**Electronic Supplementary Material:**

The online version of this article (doi:10.1007/s10953-012-9875-7) contains supplementary material, which is available to authorized users.

## Introduction

Ionic liquids (ILs) are a new class of non-molecular ionic compounds with interesting properties. ILs are usually composed of bulky and asymmetric nitrogen or phosphorus, or imidazolium, or pyridinium, or pyrrolidinium, or piperidinium containing cations paired with organic or inorganic counter anions. These compounds possess many advantages over traditional organic solvents due to their negligible vapor pressure, wide liquid ranges, tunable viscosities and often very high thermal stability [1–3]. Although thermodynamic studies on binary mixtures of thiocyanate-based ILs with various solvents are available in the literature [4–11], there are few reported densities, viscosities, or surface tensions for the binary mixtures of thiocyanate based ILs with alcohols [12–16] or water [17]. For ILs the relationship between volume, viscosity and temperature is one of the most fundamental and useful from both a theoretical and practical standpoint. These data are important for efficient design of chemical products and processes. Generally, a mixtures of water and a water-miscible IL might provide attractive alternative solvents for organic compounds.

Recently, a study of solutions of 1-ethyl-3-methylimidazolium thiocyanate, [EMIM][SCN], with water has been presented by our laboratory [9]. The excess molar volumes, $V_{\mathrm{m}}^{\mathrm{E}}$ were positive for the entire composition range and at all temperatures. This was attributed to the disruption of H-bonds of the two compounds, which makes positive contributions to $V_{\mathrm{m}}^{\mathrm{E}}$. The values of $V_{\mathrm{m}}^{\mathrm{E}}$ of a mixture formed from two self-associated (H-bonded) substances is the result of a number of effects which may contribute terms differing in sign. Specific interactions between unlike molecules and the packing effect give negative contributions to $V_{\mathrm{m}}^{\mathrm{E}}$. The $V_{\mathrm{m}}^{\mathrm{E}}$ values are negative for all mixtures of {[BMIM][SCN] + an alcohol (methanol, or ethanol, or 1-butanol) [12], or (1-butanol, 1-pentanol, 1-hexanol) [13], or (1-heptanol, 1-octanol, 1-nonanol, and 1-decanol)} [14]. Less negative excess molar volumes were obtained by lengthening of the alkyl chain of the alcohol.

The density and viscosity are affected by the nature of anion, cation, and substituents on the cation and anion of the imidazolium-based ionic liquids. Longer alkyl chains on the cation and/or anion usually gave rise to lower densities [16, 18].

Thus we report here measurements of the density and viscosity of four mixtures with water at various temperatures in range (298.15–348.15) K and ambient pressure for ILs with the same anion and the same substituents (butyl- and methyl-) on the different cations: imidazolium, pyridinium, pyrrolidinium and piperidinium. The temperature dependences of both properties are analyzed and correlated. Excess molar volumes and viscosity deviations, calculated from the experimental points, are also correlated. This information may be important for new separation technologies.

Experimental procedures and results for pure compounds and binary systems of IL with water have been tabulated in detail and then briefly discussed and interpreted below. Thus, the characteristics investigated here includes the effect of the structure (ring) of the cation on density and viscosity as a function of temperature and composition.

## Experimental

### Materials

The ionic liquid 1-butyl-3-methylimidazolium thiocyanate, [BMIM][SCN], had a purity of >0.98 mass fraction and was supplied by Fluka; 1-butyl-4-methylpyridinium thiocyanate, [BMPy][SCN], 1-butyl-1-methylpyrrolidinium thiocyanate, [BMPYR][SCN], and 1-butyl-1-methylpiperidinium thiocyanate [BMPIP][SCN] had purities of >0.99 mass fraction and were purchased from Liquid Technologies (Iolitec GmbH&Co. KG, Denzlingen, Germany), synthesized on request. Structures of the investigated ionic liquids are presented in Table 1. The ionic liquids were further purified by subjecting them to a very low pressure of about 5×10^−3^ Pa at about 300 K for approximately 10 hours. This procedure removed any volatile chemicals and water from the ionic liquid. Doubly distilled and degassed water was used in the volumetric measurements. Basic properties of the ionic liquids are listed in Table 2.

**Table 1:** Structures of ionic liquids

Structure	Name, abbreviation
	1-Butyl-3-methylimidazolium thiocyanate, [BMIM][SCN]
	1-Butyl-4-methylpyridinium thiocyanate, [BMPy][SCN]
	1-Butyl-1-methylpyrrolidinium thiocyanate, [BMPYR][SCN]
	1-Butyl-1-methylpiperidinium thiocyanate, [BMPIP][SCN]

**Table 2:** Physical properties of thiocyanate-based ionic liquids

	*M* (g⋅mol^−1^)	$V_{\mathrm{m}}^{298.15}$ (cm^3^⋅mol^−1^)	$\rho _{\exp}^{298.15}$ (g⋅cm^−3^)	$\rho _{\exp} ^{298.15}$ (g⋅cm^−3^)^a^	*η* ^298.15^ (mPa⋅s)
[BMIM][SCN]	197.3	184.4	1.06979	1.06967	51.7
[BMPy][SCN]	208.2	196.2	1.06127	1.06127	85.7
[BMPYR][SCN]	200.4	195.5	1.02477	1.03039	109.5
[BMPIP][SCN]^b^	214.4	210.3			264.5

^a^Interpolated from Ref. [17]

^b^At *T*=318.15 K

### Water Content

The water content of the pure ILs was determined by Karl–Fisher titration (method TitroLine KF) before mixing with water. Samples of ILs were dissolved in methanol and titrated with steps of 2.5 μL. The analysis showed that the water contents in the ILs were <210 ppm.

### Density Measurements

The densities of all pure ILs and water as well as their binary mixtures were measured using an Anton Paar GmbH 4500 vibrating-tube densimeter (Graz, Austria), thermostated at the different temperatures. Two integrated Pt 100 platinum thermometers provided good precision in temperature control internally (*T*±0.01 K). The densimeter includes an automatic correction for the viscosity of the sample. The apparatus is precise to within 1×10^−5^ g⋅cm^−3^, and the uncertainty of the measurements was estimated to be better than ±5×10^−5^ g⋅cm^−3^. The densimeter’s calibration was performed at atmospheric pressure using doubly distilled and degassed water, specially purified benzene (CHEMIPAN, Poland 0.999) and dried air. Mixtures were prepared by weighing; the error in mole fraction being estimated as less than 5×10^−4^. The densities of the pure ILs [BMIM][SCN], [BMPy][SCN] and [BMPYR][SCN] are in satisfactory agreement with the literature values, as is shown in Table 2. The uncertainty of the excess molar volumes depend on the uncertainties of the density measurements and was assumed to be ±0.0005 cm^3^⋅mol^−1^. The densities of water, the ILs and their mixtures are tabulated in Tables 3, 4, 5, 6.

**Table 3:** Experimental density (*ρ*), excess molar volume ($V_{\mathrm{m}}^{\mathrm{E}}$), dynamic viscosity (*η*), viscosity deviation (Δ*η*), isobaric expansivities (*α*) and excess isobaric expansivities (*α* ^E^) for the {[BMIM][SCN] (1) + water (2)} binary system

*x*_1_	*T* (K)					
	298.15	308.15	318.15	328.15	338.15	348.15
	*ρ* (g⋅cm^−3^)					
1.0000	1.06979	1.06389	1.05802	1.05219	1.04643	1.04070
0.9002	1.06911	1.06315	1.05725	1.05140	1.04556	1.03979
0.7993	1.06831	1.06232	1.05637	1.05046	1.04461	1.03878
0.6831	1.06708	1.06103	1.05502	1.04902	1.04308	1.03716
0.5654	1.06533	1.05918	1.05308	1.04699	1.04091	1.03488
0.4704	1.06329	1.05705	1.05083	1.04464	1.03845	1.03226
0.3574	1.05954	1.05318	1.04680	1.04042	1.03404	1.02764
0.2799	1.05564	1.04917	1.04268	1.03616	1.02960	1.02299
0.2051	1.04993	1.04342	1.03682	1.03017	1.02345	1.01666
0.1267	1.04000	1.03364	1.02712	1.02045	1.01363	1.00674
0.0556	1.02434	1.01779	1.01189	1.00566	0.99912	0.99262
0.0000	0.99704	0.99403	0.99020	0.98569	0.98030	0.97475
	$V_{\mathrm{m}}^{\mathrm{E}}\ (\mbox{cm}^{3}{\cdot}\mbox{mol}^{-1})$					
1.0000	0.0000	0.0000	0.0000	0.0000	0.0000	0.0000
0.9002	−0.0159	−0.0014	0.0071	0.0129	0.0268	0.0340
0.7993	−0.0378	−0.0149	0.0040	0.0206	0.0354	0.0513
0.6831	−0.0560	−0.0213	0.0079	0.0374	0.0631	0.0895
0.5654	−0.0669	−0.0183	0.0224	0.0614	0.1002	0.1361
0.4704	−0.0661	−0.0080	0.0440	0.0910	0.1363	0.1836
0.3574	−0.0468	0.0204	0.0827	0.1409	0.1943	0.2505
0.2799	−0.0305	0.0422	0.1084	0.1708	0.2289	0.2909
0.2051	−0.0083	0.0640	0.1315	0.1945	0.2522	0.3136
0.1267	0.0176	0.0813	0.1398	0.1954	0.2455	0.2976
0.0556	0.0271	0.0677	0.1046	0.1399	0.1697	0.2535
0.0000	0.0000	0.0000	0.0000	0.0000	0.0000	0.0000
	*η* (mPa⋅s)					
1.0000	51.74	34.87	24.19	16.70	12.73	9.98
0.9002	40.19	26.93	19.14	14.22	10.98	8.73
0.7993	32.97	22.49	16.17	12.20	9.53	7.61
0.6831	25.93	18.06	13.24	10.12	7.96	6.40
0.5654	19.54	13.91	10.40	8.04	6.37	5.14
0.4704	15.17	10.96	8.29	6.44	5.12	4.16
0.3574	10.80	7.95	6.05	4.71	3.76	3.10
0.2799	8.27	6.14	4.66	3.65	2.96	2.48
0.2051	5.95	4.36	3.33	2.67	2.23	1.86
0.1267	3.75	2.84	2.23	1.82	1.51	1.28
0.0556	2.02	1.58	1.27	1.05	0.90	0.78
0.0000	0.890	0.740	0.630	0.560	0.500	0.46
	Δ*η* (mPa⋅s)					
1.0000	0.00	0.00	0.00	0.00	0.00	0.00
0.9002	−6.48	−4.53	−2.70	−0.87	−0.53	−0.30
0.7993	−8.56	−5.53	−3.29	−1.26	−0.75	−0.46
0.6831	−9.70	−5.99	−3.48	−1.47	−0.89	−0.56
0.5654	−10.10	−6.13	−3.55	−1.65	−1.04	−0.70
0.4704	−9.64	−5.83	−3.42	−1.71	−1.13	−0.78
0.3574	−8.26	−4.99	−3.00	−1.62	−1.11	−0.76
0.2799	−6.85	−4.15	−2.56	−1.43	−0.96	−0.64
0.2051	−5.37	−3.38	−2.13	−1.20	−0.78	−0.55
0.1267	−3.58	−2.22	−1.39	−0.78	−0.54	−0.39
0.0556	−1.70	−1.06	−0.67	−0.41	−0.28	−0.21
0.0000	0.00	0.00	0.00	0.00	0.00	0.00
	10^4^ *α* (K^−1^)					
1.0000	5.55	5.53	5.52	5.51	5.49	5.48
0.9002	5.54	5.54	5.53	5.53	5.52	5.51
0.7993	5.60	5.60	5.61	5.61	5.61	5.61
0.6831	5.68	5.69	5.71	5.72	5.74	5.75
0.5654	5.73	5.77	5.80	5.83	5.87	5.90
0.4704	5.78	5.83	5.89	5.94	5.99	6.04
0.3574	5.87	5.96	6.04	6.13	6.21	6.30
0.2799	5.96	6.08	6.20	6.32	6.44	6.56
0.2051	6.04	6.21	6.37	6.54	6.71	6.89
0.1267	5.93	6.19	6.45	6.70	6.96	7.23
0.0556	5.15	5.56	5.98	6.40	6.82	7.25
0.0000	2.81	3.50	4.19	4.90	5.61	6.34
	10^4^ *α* ^E^ (K^−1^)					
1.0000	0.00	0.00	0.00	0.00	0.00	0.00
0.9002	0.03	0.02	0.02	0.02	0.02	0.02
0.7993	0.12	0.12	0.12	0.12	0.11	0.11
0.6831	0.25	0.24	0.24	0.24	0.24	0.23
0.5654	0.38	0.37	0.37	0.37	0.36	0.36
0.4704	0.51	0.50	0.50	0.49	0.48	0.48
0.3574	0.73	0.73	0.72	0.71	0.70	0.69
0.2799	0.97	0.96	0.95	0.94	0.92	0.91
0.2051	1.25	1.23	1.22	1.20	1.19	1.17
0.1267	1.49	1.48	1.46	1.44	1.42	1.40
0.0556	1.31	1.30	1.29	1.27	1.25	1.23
0.0000	0.00	0.00	0.00	0.00	0.00	0.00

**Table 4:** Experimental density (*ρ*), excess molar volume ($V_{\mathrm{m}}^{\mathrm{E}}$), dynamic viscosity (*η*), viscosity deviation (Δ*η*), isobaric expansivities (*α*) and excess isobaric expansivities (*α* ^E^) for the {[BMPy][SCN] (1) + water (2)} binary system

*x*_1_	*T* (K)					
	298.15	308.15	318.15	328.15	338.15	348.15
	*ρ* (g⋅cm^−3^)					
1.0000	1.06127	1.05558	1.04990	1.04426	1.03866	1.03309
0.9436	1.06099	1.05528	1.04958	1.04390	1.03828	1.03268
0.8345	1.06035	1.05457	1.04882	1.04308	1.03738	1.03172
0.7220	1.05947	1.05361	1.04778	1.04197	1.03619	1.03044
0.6203	1.05834	1.05239	1.04647	1.04056	1.03469	1.02883
0.5372	1.05714	1.05110	1.04508	1.03909	1.03310	1.02712
0.4331	1.05494	1.04876	1.04259	1.03642	1.03027	1.02411
0.3636	1.05285	1.04656	1.04027	1.03397	1.02766	1.02136
0.3001	1.05020	1.04384	1.03743	1.03100	1.02440	1.01807
0.2377	1.04667	1.04018	1.03365	1.02706	1.02043	1.01375
0.1667	1.04067	1.03413	1.02751	1.02080	1.01402	1.00712
0.0972	1.03068	1.02444	1.01802	1.01078	1.00456	0.99756
0.0430	1.01645	1.01120	1.00550	0.99744	0.99212	0.98670
0.0286	1.01103	1.00629	1.00101	0.99305	0.98656	0.98173
0.0076	1.00126	0.99644	0.99035	0.98581	0.98065	0.97524
0.0000	0.99704	0.99403	0.99020	0.98569	0.98030	0.97475
	$V_{\mathrm{m}}^{\mathrm{E}}\ (\mbox{cm}^{3}{\cdot}\mbox{mol}^{-1})$					
1.0000	0.0000	0.0000	0.0000	0.0000	0.0000	0.0000
0.9436	−0.0126	−0.0065	−0.0010	0.0074	0.0113	0.0170
0.8345	−0.0366	−0.0146	0.0021	0.0218	0.0390	0.0544
0.7220	−0.0554	−0.0187	0.0117	0.0419	0.0694	0.0965
0.6203	−0.0605	−0.0108	0.0317	0.0738	0.1103	0.1487
0.5372	−0.0637	−0.0038	0.0492	0.0982	0.1449	0.1932
0.4331	−0.0525	0.0192	0.0837	0.1459	0.2015	0.2602
0.3636	−0.0392	0.0391	0.1101	0.1782	0.2411	0.3047
0.3001	−0.0197	0.0600	0.1352	0.2069	0.2832	0.3418
0.2377	−0.0029	0.0809	0.1578	0.2322	0.3001	0.3716
0.1667	0.0162	0.0965	0.1700	0.2401	0.3029	0.3715
0.0972	0.0333	0.0968	0.1546	0.2321	0.2605	0.3160
0.0430	0.0419	0.0779	0.1118	0.1966	0.1950	0.1934
0.0286	0.0356	0.0615	0.0858	0.1621	0.1650	0.1735
0.0076	0.0143	0.0459	0.0586	0.1082	0.0987	0.0821
0.0000	0.0000	0.0000	0.0000	0.0000	0.0000	0.0000
	*η* (mPa⋅s)					
1.0000	85.69	51.75	33.88	23.51	17.28	12.86
0.9436	74.91	45.99	30.43	21.32	15.54	11.74
0.8345	57.59	36.38	24.60	17.46	12.90	9.89
0.7220	43.08	28.02	19.33	13.95	10.49	8.18
0.6203	31.26	20.86	14.68	10.87	8.38	6.62
0.5372	24.82	16.80	12.14	9.17	7.17	5.73
0.4331	17.83	12.47	9.17	7.02	5.54	4.45
0.3636	14.06	10.02	7.48	5.78	4.58	3.69
0.2377	8.60	6.30	4.78	3.70	2.94	2.44
0.1667	6.04	4.45	3.37	2.66	2.08	1.62
0.0972	3.65	2.74	2.13	1.71	1.41	1.24
0.0430	1.98	1.53	1.23	1.01	0.86	0.75
0.0286	1.58	1.24	1.01	0.84	0.73	0.64
0.0076	1.07	0.87	0.73	0.63	0.57	0.52
0.0000	0.89	0.74	0.63	0.56	0.50	0.43
	Δ*η* (mPa⋅s)					
1.0000	0.00	0.00	0.00	0.00	0.00	0.00
0.9436	−5.99	−2.88	−1.58	−0.90	−0.55	−0.42
0.8345	−14.07	−6.93	−3.78	−2.25	−1.39	−0.92
0.7220	−19.04	−9.55	−5.31	−3.18	−1.94	−1.24
0.6203	−22.23	−11.52	−6.58	−3.93	−2.38	−1.53
0.5372	−21.63	−11.35	−6.35	−3.72	−2.21	−1.39
0.4331	−19.78	−10.36	−5.86	−3.48	−2.11	−1.38
0.3636	−17.66	−9.27	−5.24	−3.12	−1.93	−1.28
0.2377	−12.44	−6.56	−3.76	−2.32	−1.49	−0.97
0.1667	−8.99	−4.79	−2.81	−1.73	−1.18	−0.91
0.0972	−5.48	−2.96	−1.73	−1.08	−0.69	−0.43
0.0430	−2.56	−1.41	−0.84	−0.54	−0.35	−0.24
0.0286	−1.73	−0.96	−0.57	−0.37	−0.24	−0.17
0.0076	−0.47	−0.26	−0.15	−0.10	−0.06	−0.03
0.0000	0.00	0.00	0.00	0.00	0.00	0.00
	10^4^ *α* (K^−1^)					
1.0000	5.40	5.39	5.38	5.38	5.38	5.38
0.9436	5.34	5.40	5.40	5.40	5.40	5.40
0.8345	5.45	5.46	5.47	5.48	5.49	5.50
0.7220	5.52	5.54	5.56	5.58	5.60	5.62
0.6203	5.58	5.62	5.65	5.68	5.71	5.74
0.5372	5.64	5.69	5.73	5.78	5.82	5.87
0.4331	5.74	5.81	5.88	5.94	6.01	6.08
0.3636	5.84	5.93	6.01	6.10	6.19	6.28
0.2377	5.95	6.06	6.17	6.28	6.39	6.51
0.1667	6.06	6.20	6.35	6.49	6.63	6.78
0.0972	6.11	6.31	6.51	6.71	6.92	7.12
0.0430	5.84	6.14	6.45	6.75	7.05	7.36
0.0286	4.92	5.37	5.83	6.28	6.75	7.21
0.0076	4.43	4.94	5.46	5.98	6.51	7.04
0.0000	3.35	3.98	4.62	5.27	5.93	6.59
	10^4^ *α* ^E^ (K^−1^)					
1.0000	0.00	0.00	0.00	0.00	0.00	0.00
0.9436	0.02	0.02	0.02	0.02	0.02	0.02
0.8345	0.10	0.11	0.11	0.10	0.10	0.10
0.7220	0.22	0.22	0.22	0.21	0.21	0.21
0.6203	0.33	0.33	0.33	0.32	0.32	0.32
0.5372	0.44	0.44	0.43	0.43	0.42	0.42
0.4331	0.63	0.63	0.62	0.61	0.61	0.60
0.3636	0.81	0.80	0.79	0.78	0.78	0.77
0.2377	1.02	1.01	1.00	0.98	0.97	0.96
0.1667	1.26	1.25	1.23	1.22	1.20	1.19
0.0972	1.54	1.52	1.50	1.48	1.46	1.44
0.0430	1.65	1.63	1.61	1.59	1.57	1.54
0.0286	1.27	1.26	1.24	1.23	1.21	1.19
0.0076	1.00	0.99	0.98	0.96	0.95	0.94
0.0000	0.35	0.34	0.34	0.34	0.33	0.33

**Table 5:** Experimental density (*ρ*), excess molar volume ($V_{\mathrm{m}}^{\mathrm{E}}$), dynamic viscosity (*η*), viscosity deviation (Δ*η*), isobaric expansivities (*α*) and excess isobaric expansivities (*α* ^E^) for the {[BMPYR][SCN] (1) + water (2)} binary system

*x*_1_	*T* (K)					
	298.15	308.15	318.15	328.15	338.15	348.15
	*ρ* (g⋅cm^−3^)					
1.0000	1.02477	1.01945	1.01421	1.00901	1.00385	0.99873
0.9147	1.02472	1.01941	1.01415	1.00893	1.00372	0.99852
0.7797	1.02470	1.01939	1.01404	1.00875	1.00346	0.99819
0.6445	1.02466	1.01920	1.01376	1.00833	1.00293	0.99754
0.4748	1.02407	1.01841	1.01274	1.00710	1.00147	0.99583
0.3366	1.02262	1.01671	1.01079	1.00486	0.99891	0.99296
0.2170	1.01966	1.01354	1.00739	1.00118	0.99511	0.98882
0.1377	1.01543	1.00936	1.00314	0.99684	0.99136	0.98479
0.0343	1.00404	0.99865	0.99334	0.98783	0.98255	0.97677
0.0000	0.99704	0.99403	0.99020	0.98569	0.98030	0.97475
	$V_{\mathrm{m}}^{\mathrm{E}}\ (\mbox{cm}^{3}{\cdot}\mbox{mol}^{-1})$					
1.0000	0.0000	0.0000	0.0000	0.0000	0.0000	0.0000
0.9147	−0.0329	−0.0314	−0.0260	−0.0215	−0.0129	−0.0027
0.7797	−0.0971	−0.0903	−0.0684	−0.0522	−0.0330	−0.0110
0.6445	−0.1597	−0.1281	−0.0938	−0.0596	−0.0295	0.0041
0.4748	−0.1871	−0.1326	−0.0766	−0.0253	0.0212	0.0719
0.3366	−0.1616	−0.0900	−0.0210	0.0446	0.1050	0.1674
0.2170	−0.1013	−0.0246	0.0471	0.1155	0.1662	0.2303
0.1377	−0.0346	0.0332	0.0978	0.1576	0.1695	0.2286
0.0343	0.0170	0.0596	0.0870	0.1080	0.1080	0.1180
0.0000	0.0000	0.0000	0.0000	0.0000	0.0000	0.0000
	*η* (mPa⋅s)					
1.0000	109.46	70.36	47.79	34.13	25.30	19.35
0.9147	91.83	59.38	40.28	28.87	21.54	16.50
0.7797	68.57	45.17	31.56	22.99	17.27	13.32
0.6445	49.28	33.17	23.48	17.26	13.08	10.21
0.4748	28.85	19.87	14.38	10.87	8.49	6.81
0.3366	17.56	12.37	9.15	7.02	5.53	4.42
0.2170	10.04	7.27	5.46	4.19	3.27	2.65
0.1377	5.90	4.31	3.23	2.54	2.02	1.67
0.0343	1.74	1.36	1.10	0.92	0.790	0.690
0.0000	0.890	0.740	0.63	0.560	0.500	0.430
	Δ*η* (mPa⋅s)					
1.0000	0.00	0.00	0.00	0.00	0.00	0.00
0.9147	−8.37	−5.04	−3.49	−2.40	−1.64	−1.24
0.7797	−16.97	−9.85	−5.84	−3.74	−2.57	−1.87
0.6445	−21.58	−12.44	−7.54	−4.94	−3.40	−2.42
0.4748	−23.59	−13.93	−8.64	−5.63	−3.79	−2.62
0.3366	−19.87	−11.80	−7.35	−4.84	−3.32	−2.40
0.2170	−14.41	−8.58	−5.40	−3.65	−2.61	−1.91
0.1377	−9.94	−6.02	−3.90	−2.64	−1.90	−1.39
0.0343	−2.88	−1.77	−1.15	−0.79	−0.56	−0.42
0.0000	0.00	0.00	0.00	0.00	0.00	0.00
	10^4^ *α* (K^−1^)					
1.0000	5.08	5.10	5.11	5.12	5.14	5.15
0.9147	5.13	5.15	5.17	5.19	5.21	5.22
0.7797	5.18	5.21	5.24	5.27	5.30	5.33
0.6445	5.25	5.29	5.34	5.38	5.43	5.47
0.4748	5.42	5.49	5.56	5.64	5.71	5.78
0.3366	5.61	5.72	5.83	5.94	6.05	6.16
0.2170	5.68	5.86	6.03	6.20	6.38	6.55
0.1377	5.51	5.76	6.01	6.26	6.52	6.78
0.0343	4.18	4.67	5.17	5.68	6.19	6.71
0.0000	2.81	3.50	4.19	4.90	5.61	6.34
	10^4^ *α* ^E^ (K^−1^)					
1.0000	0.00	0.00	0.00	0.00	0.00	0.00
0.9147	0.07	0.07	0.07	0.07	0.07	0.06
0.7797	0.16	0.15	0.15	0.15	0.15	0.15
0.6445	0.28	0.28	0.27	0.27	0.27	0.26
0.4748	0.55	0.55	0.54	0.53	0.53	0.52
0.3366	0.88	0.87	0.86	0.85	0.84	0.83
0.2170	1.17	1.16	1.15	1.13	1.12	1.11
0.1377	1.26	1.25	1.24	1.22	1.21	1.19
0.0343	0.74	0.73	0.72	0.72	0.71	0.70
0.0000	0.00	0.00	0.00	0.00	0.00	0.00

**Table 6:** Experimental density (*ρ*), excess molar volume ($V_{\mathrm{m}}^{\mathrm{E}}$), dynamic viscosity (*η*), viscosity deviation (Δ*η*), isobaric expansivities (*α*) and excess isobaric expansivities (*α* ^E^) for the {[BMPIP][SCN] (1) + water (2)} binary system

*x*_1_	*T* (K)			
	318.15	328.15	338.15	348.15
	*ρ* (g⋅cm^−3^)			
1.0000	1.01953	1.01417	1.00913	1.00403
0.8954	1.01946	1.01406	1.00897	1.00378
0.7226	1.01903	1.01362	1.00835	1.00302
0.6259	1.01859	1.01315	1.00764	1.00219
0.4889	1.01757	1.01191	1.00624	1.00058
0.3698	1.01593	1.01004	1.00413	0.99820
0.2828	1.01391	1.00779	1.00164	0.99546
0.1752	1.00924	1.00286	0.99641	0.98986
0.0649	0.99924	0.99311	0.98732	0.98151
0.0000	0.99020	0.98569	0.98030	0.97475
	$V_{\mathrm{m}}^{\mathrm{E}}\ (\mathrm{cm}^{3} {\cdot}\mathrm{mol}^{ - 1})$			
1.0000	0.0000	0.0000	0.0000	0.0000
0.8954	−0.0417	−0.0330	−0.0245	−0.0083
0.7226	−0.0683	−0.0569	−0.0231	0.0108
0.6259	−0.0683	−0.0522	0.0100	0.0560
0.4889	−0.0522	−0.0113	0.0561	0.1160
0.3698	−0.0149	0.0419	0.1163	0.1875
0.2828	0.0244	0.0910	0.1684	0.2440
0.1752	0.0924	0.1594	0.2299	0.3027
0.0649	0.1231	0.1631	0.1822	0.1988
0.0000	0.0000	0.0000	0.0000	0.0000
	*η* (mPa⋅s)			
1.0000	264.54	151.63	93.55	60.53
0.7226	94.87	59.36	39.62	27.83
0.6259	57.30	37.20	25.60	18.51
0.4889	33.00	22.33	15.96	11.92
0.3698	19.13	13.48	9.94	7.60
0.2828	12.13	8.79	6.60	5.08
0.1752	6.02	4.47	3.41	2.71
0.0649	1.97	1.57	1.28	1.07
0.0000	0.630	0.56	0.500	0.430
	Δ*η* (mPa⋅s)			
1.0000	0.00	0.00	0.00	0.00
0.7226	−96.45	−50.36	−28.12	−16.03
0.6259	−108.50	−57.91	−33.14	−19.54
0.4889	−96.64	−52.08	−30.03	−17.89
0.3698	−79.09	−42.95	−24.96	−15.06
0.2828	−63.14	−34.50	−20.21	−12.34
0.1752	−40.85	−22.56	−13.39	−8.25
0.0649	−15.78	−8.79	−5.25	−3.25
0.0000	0.00	0.00	0.00	0.00
	10^4^ *α* (K^−1^)			
1.0000	5.27	5.15	5.04	4.92
0.8954	5.31	5.20	5.09	4.98
0.7226	5.38	5.29	5.20	5.10
0.6259	5.45	5.37	5.29	5.22
0.4889	5.62	5.57	5.52	5.47
0.3698	5.85	5.83	5.82	5.80
0.2828	6.05	6.07	6.09	6.12
0.1752	6.22	6.33	6.44	6.55
0.0649	5.83	6.16	6.49	6.82
0.0000	4.19	4.90	5.61	6.34
	10^4^ *α* ^E^ (K^−1^)			
1.0000	0.00	0.00	0.00	0.00
0.8954	0.05	0.05	0.05	0.05
0.7226	0.14	0.14	0.14	0.14
0.6259	0.23	0.23	0.23	0.23
0.4889	0.44	0.44	0.43	0.43
0.3698	0.72	0.71	0.71	0.70
0.2828	0.97	0.96	0.95	0.94
0.1752	1.27	1.25	1.24	1.22
0.0649	1.16	1.15	1.13	1.12
0.0000	0.00	0.00	0.00	0.00

### Viscosity Measurements

Viscosity measurements were carried out in an Anton Paar BmbH AMVn (Graz, Austria) programmable rheometer, with a nominal uncertainty of ±0.1 % and reproducibility <0.05 % for viscosities from 0.3 mPa⋅s to 2500 mPa⋅s. Temperature was controlled internally with a precision of ±0.01 K in a range from 298.15 K to 348.15 K. The diameter of the capillary was 1.6 mm for viscosities from 0.3 to 10 mPa⋅s and the diameter of the ball was 1.5 mm; the second was 1.8 mm for viscosities from 2.5 to 70 mPa⋅s and the diameter of the ball was 1.5 mm; and the third one was 3.0 mm for viscosities from 20 to 230 mPa⋅s and the diameter of the ball was 2.5 mm.

### DSC Measurements

Basic thermal characteristics of the ionic liquid, [BMPy][SCN], i.e. glass transition temperature (*T*_g,1_) and change of heat capacity at the glass transition temperature, *T*_g,1_ (Δ*C*p_(g),1_), were measured using a differential scanning microcalorimetry technique at the 5 K⋅min^−1^ scan rate with the power sensitivity of 16 mJ⋅s^−1^ and with a recorder sensitivity of 5 mV. The applied scan rate was 10 K⋅min^−1^, with power and recorder sensitivities of 16 mJ⋅s^−1^ and 5 mV, respectively. The apparatus (Thermal Analysis Q200, USA with Liquid Nitrogen Cooling System) was calibrated with a 0.999999 mol fraction purity indium sample. The calorimetric accuracy was ±3%. The thermophysical properties (average over three scans) are shown in the DSC diagram (GRS.1 in the Electronic Supplementary Material (ESM)). The average value of the glass transition temperature *T*_g,1_ was (199.9±0.1) K with Δ*Cp*_(g),1_ of (210±3) J⋅mol^−1^⋅K^−1^ (average over three scans).

### Decomposition of the Compounds

**Fig. 1:**
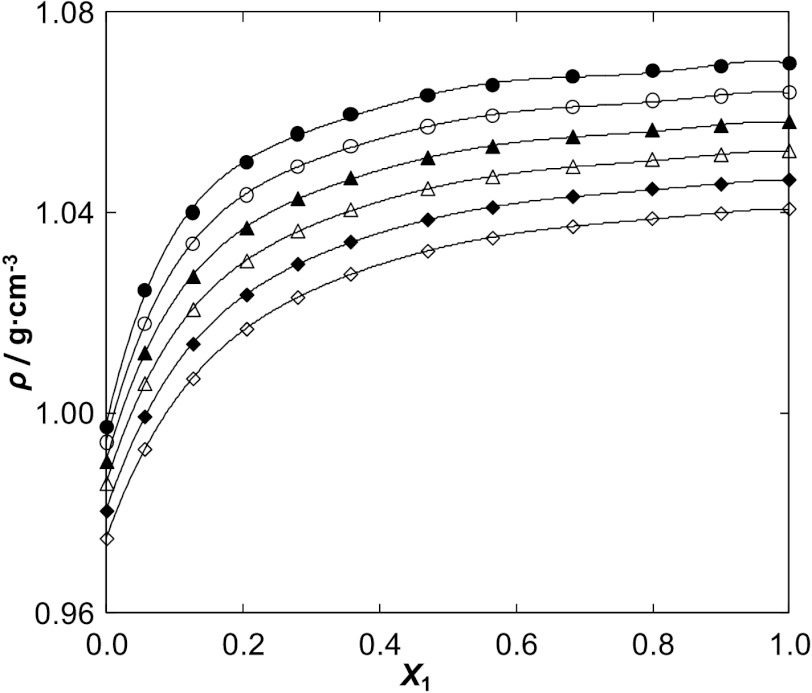
Density *ρ* for the {[BMIM][SCN] (1) + water (2)} system as a function of the mole fraction of the IL at different temperatures: ●, 298.15 K; ○, 308.15 K; ▲, 318.15 K; △, 328.15 K, ◇, 338.15 K; ◆, 348.15 K

Simultaneous TG/DTA experiments was performed using a Q600 TA Instruments. In general, runs were carried out using matched labyrinth platinic crucibles with Al_2_O_3_ in the reference pan. The crucible design hampered the migration of the volatile decomposition products thereby reducing the rate of gas evolution and, in turn, increasing the contact time of the reactants. All the TG/DTA curves were obtained at 5 K⋅min^−1^ heating rate with a dynamic nitrogen atmosphere (flow rate 20 dm^3^⋅h^−1^). The scanning rate was provided over the temperature range (200–770) K. The temperature control was (*T*±0.001) K. The temperature of decomposition is presented in Fig. 1S in the ESM. The decomposition is observed at temperature (546±3) K.

## Results and Discussion

### Effect of Temperature on Density and Viscosity

The experimental data of density, *ρ*, and dynamic viscosity, *η*, versus *x*_1_, the mole fraction of the {IL (1) + water (2)}, at different temperatures are listed in Tables 3, 4, 5, and 6. The densities and viscosities, as usual in such a systems, are higher for the IL than for water and decrease with an increasing amount of water. The densities of the ionic liquids at *T*=318.15 K are 1.05802, 1.04990, 1.01421 and 1.01953 g⋅cm^−3^ for [BMIM][SCN], [BMPy][SCN], [BMPYR][SCN] and [BMPIP][SCN], respectively. Thus the lowest density was observed for [BMPYR][SCN]. The dynamic viscosities at *T*=318.15 K are 24.19, 33.88, 47.79, and 264.54 mPa⋅s for [BMIM][SCN], [BMPy][SCN], [BMPYR][SCN] and [BMPIP][SCN], respectively. It can be observed that there is a huge difference between the values for first three of ILs and for [BMPIP][SCN]. As always, both density and viscosity decrease with an increase of temperature. We found no previous data for these systems as a function of temperature for comparison. The experimental data for [BMPIP][SCN] were measured from temperature *T*=318.15 K to 348.15 K, because of the high melting temperature of this IL (304.32 K [19]).

**Fig. 2:**
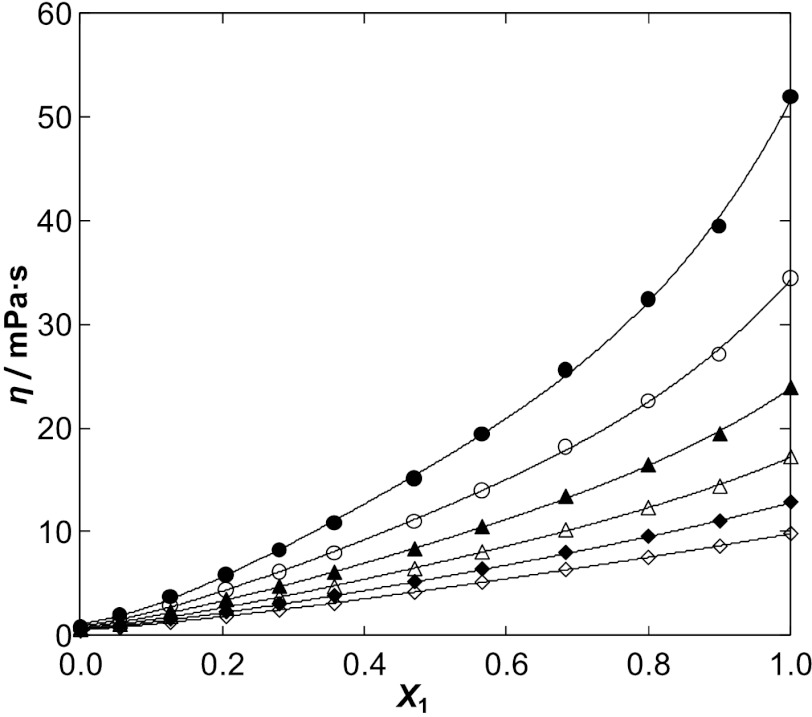
Dynamic viscosity *η* for the {[BMIM][SCN] (1) + water (2)} system as a function of the mole fraction of the IL at different temperatures: ●, 298.15 K; ○, 308.15 K; ▲, 318.15 K; △, 328.15 K; ◇, 338.15 K; ◆, 348.15 K

Experimental densities at ambient pressure, investigated in this work are shown in Fig. 2S a–d in the ESM. As already mentioned, the density decreases as both temperature and water concentration in the system increase. The dependency on temperature was correlated with a second-order polynomial. Fitting parameters are listed in Table 1S in the ESM for pure ILs and in Table 2S in the ESM for the mixtures with water.

**Fig. 3:**
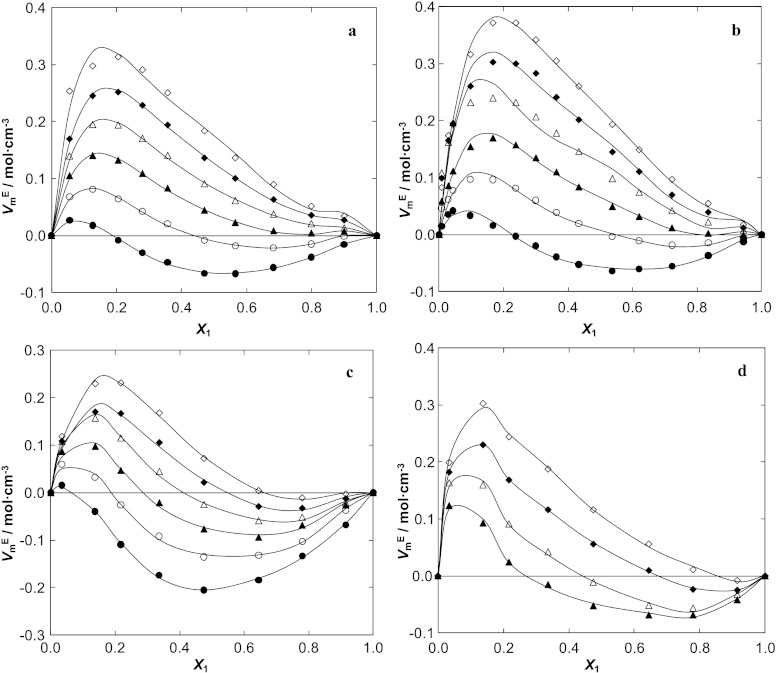
Excess molar volume $V_{\mathrm{m}}^{\mathrm{E}}$ versus *x*_1_ for the (**a**) {[BMIM][SCN] (1) + water (2)}, (**b**) {[BMPy][SCN] (1) + water (2)}, (**c**) {[BMPYR][SCN] (1) + water (2)}, (**d**) {[BMPIP][SCN] (1) + water (2)} binary systems at different temperatures: ●, 298.15 K; ○, 308.15 K; ▲, 318.15 K; △, 328.15 K; ◆, 338.15 K; ◇, 348.15 K. The *solid lines* represent the corresponding correlations by the Redlich–Kister equation

The effect of temperature on viscosity is similar. Figure 3S a–d depicts the experimental dynamic viscosities for the four binary systems studied, as a function of temperature, for different compositions together with the well-known Vogel–Fulcher–Tammann, VFT equation [20–22]. This equation has been used to correlate variations of viscosity with temperature in measured systems:

1 $$ \eta = AT^{0.5}\exp\biggl(\frac{B}{T - T_{0}}\biggr) $$

The fitting parameters, determined empirically, are in general *A*,*B* and *T*_0_ when the linear relation is observed between logarithmic value of *ηT* ^0.5^ and (*T*−*T*_0_)^−1^ according to Eq. 2 with three adjustable parameters. However, for glass-forming liquids, the refined value for *T*_0_ (called the ideal transition temperature) can be found from the glass transition temperature. The difference between *T*_0_ and *T*_tr(g)_ is approximately 50–60 K [23]. It is known that *T*_0_ is lower than that observed from the DSC glass transition temperature. For the ILs under study, the temperature of glass transition, determined by DSC are 181.6 K [5], 199.9 K [this work], 180.7 K [24], and 201.3 K [19], for [BMIM][SCN], [BMPy][SCN], [BMPYR][SCN] and [BMPIP][SCN], respectively. The ideal transition temperature, taken for the calculations, was 50 K lower than the glass transition temperature.

One value of parameter *T*_0_ was used for all the solutions with water for various temperatures and concentrations, because Δ*T*_0_, as discussed by many authors, is of the order of about 10 K. The values of *A* and *B* together with root-mean square deviations, *σ*, are presented in Tables 3S and 4S in the ESM.

The VFT equation suitably correlates the viscosities of pure IL and the viscosities of the mixtures for the binary systems throughout the composition range. The parameters *A* and *B* in Eq. 2 change smoothly with composition for all systems. These parameters are strongly sensitive to the choice of *T*_0_.

### Effect of Composition on Density and Viscosity

**Fig. 4:**
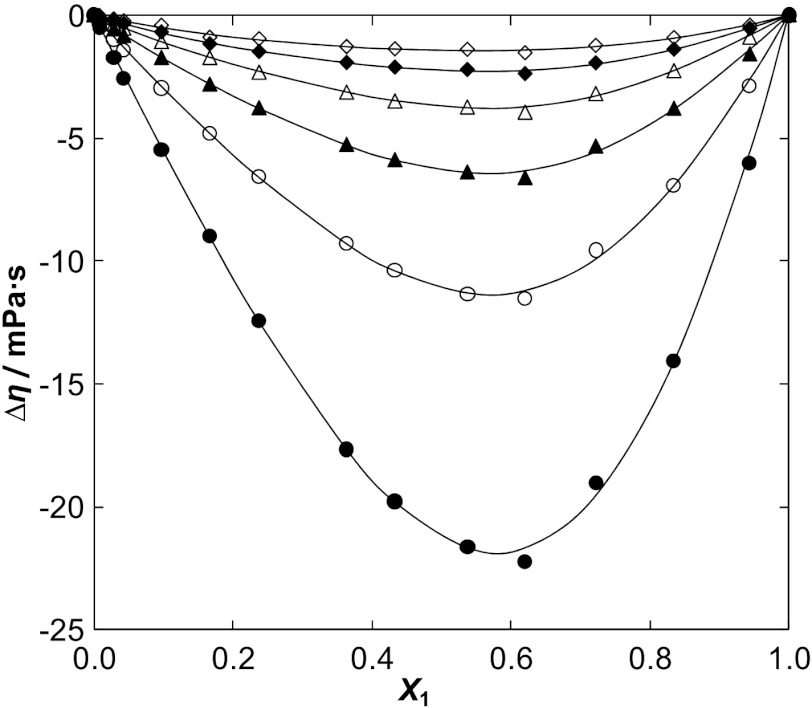
Viscosity deviation Δ*η* for the {[BMPy][SCN] (1) + water (2)} binary system at different temperatures: ●, 298.15 K; ○, 308.15 K; ▲, 318.15 K; △, 328.15 K; ◇, 338.15 K; ◆, 348.15 K. The *solid lines* represent the corresponding correlations by the Redlich–Kister equation

The densities of the IL decrease with an increase of water content. The character of the changes is presented in Fig. 1 for [BMIM][SCN] as an example, and in Fig. 4S a–c in the ESM for the former ILs together with solid lines calculated by the polynomial. The parameters of correlation are shown in Table 5S in the ESM.

**Fig. 5:**
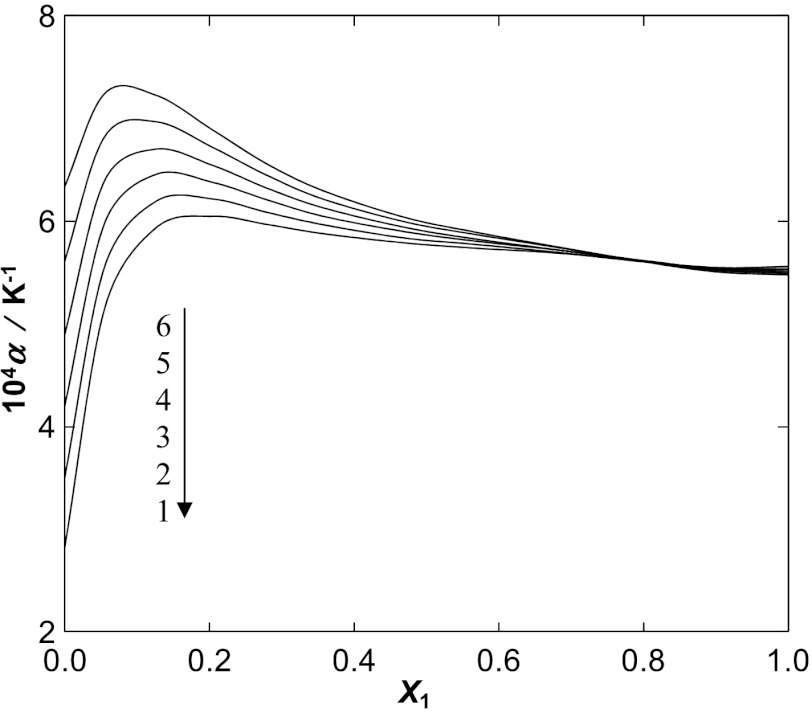
Plot of isobaric expansivity *α* of the {[BMIM][SCN] (1) + water (2)} binary system against mole fraction *x*_1_ at different temperatures: (**1**) 298.15 K, (**2**) 308.15 K, (**3**) 318.15 K, (**4**) 328.15 K, (**5**) 338.15 K, (**6**) 348.15 K

The character of the of the dynamic viscosities with composition is presented in Fig. 2 for [BMIM][SCN] as an example and in Fig. 5S a–c in the ESM for the former ILs, together with solid lines calculated by the polynomial. The parameters of the correlations are shown in Table 6S in the ESM. The observed decrease of viscosity with an increase of water content is particularly strong at low temperatures. At higher temperatures, the differences between the viscosities of pure IL and water are much lower. The interaction between the cation/anion of the IL and water increases for higher concentrations of water in the solution and the differences between viscosities of the pure IL and water are much lower for these mixtures. This weakening of the strong hydrogen bonding interactions between cation and anion of the IL leads to a higher mobility of the ions and a lower viscosity of the mixture.

The values of excess molar volumes, $V_{\mathrm{m}}^{\mathrm{E}}$, of the mixtures were calculated from the densities of binary mixtures. Systems formed from two self-associated (hydrogen-bonded) substances can produce a number of effects, which may contribute terms to $V_{\mathrm{m}}^{\mathrm{E}}$ differing in sign. Disruption of H-bonds makes a positive contribution, but specific interactions make negative contributions to $V_{\mathrm{m}}^{\mathrm{E}}$. The free volume effect, which depends on differences in the characteristic pressures and temperatures of the components, makes a negative contribution. Packing effects or conformational changes of the molecules in the mixtures are more difficult to categorize. However, interstitial accommodation and the effect of condensation give further negative contributions.

Experimental excess molar volumes, $V_{\mathrm{m}}^{\mathrm{E}}$, of {IL (1) + water (2)} mixtures are listed in Tables 3, 4, 5, 6. The data were correlated by the smoothing Redlich–Kister equation:

2 $$ V_{\mathrm{m}}^{\mathrm{E}} = x_{1}(1 - x_{1})\sum _{i = 1}^{4} A_{{i}}(T) (2x_{1} - 1)^{i - 1} $$

with

3 $$ A_{{i}}(T) = b_{{i}} + c_{{i}}T $$

where *x*_1_ is the mole fraction of the IL and $V_{\mathrm{m}}^{\mathrm{E}}\ ( \mbox{cm}^{3}{\cdot}\mbox{mol}^{-1})$ is the molar excess volume. The values of the parameters (*A*_*i*_) have been determined using the method of least-squares. The fitting parameters are summarized in Table 7. The values of standard deviations for this correlation are listed in Table 7S in the ESM.

**Table 7:** Coefficients of the Redlich–Kister equation for the correlation^a^ of the excess molar volume $V_{\mathrm{m}}^{\mathrm{E}}\ (\mathrm{cm}^{3} {\cdot}\mathrm{mol}^{ - 1})$ of the binary systems {IL (1) + water (2)}

*i*	*b*_*i*_	10^2^ ***⋅*** *c*_*i*_
[BMIM][SCN] (1) + water (2)		
1	−5.257	1.664
2	5.720	−1.921
3	−3.860	1.430
4	0.468	−0.259
[BMPy][SCN] (1) + water (2)		
1	−6.615	2.142
2	5.497	−1.869
3	−7.196	2.588
4	6.952	−2.563
[BMPYR][SCN] (1) + water (2)		
1	−7.048	2.093
2	7.070	−2.323
3	−4.294	1.660
4	−2.095	0.363
[BMPIP][SCN] (1) + water (2)		
1	−6.306	1.913
2	8.296	−2.690
3	−5.681	2.077
4	−0.715	−0.197

^a^Equation used: $V_{\mathrm{m}}^{\mathrm{E}}\ (\mathrm{cm}^{3} {\cdot}\mathrm{mol}^{ - 1}) = x_{1}(1 - x_{1})\sum_{i = 1}^{4} A_{i}(T)(2x_{1} - 1)^{i - 1}$ where *A*_*i*_(*T*)=*b*_*i*_+*c*_*i*_(*T*/*K*)

The variation of $V_{\mathrm{m}}^{\mathrm{E}}$ with mole fraction, *x*_1_, as well as the Redlich–Kister fits are shown in Fig. 3 a–d. The graphs of $V_{\mathrm{m}}^{\mathrm{E}}$ indicate that for the remaining systems studied {IL (1) + water (2)}, the $V_{\mathrm{m}}^{\mathrm{E}}$ values are S-shaped and mostly positive at the higher temperatures. The negative deviations from ideality are observed mainly at low temperatures and for the IL-rich compositions. Graphs also depict the strong unsymmetrical behavior of these excess molar volumes with composition. The maximum values of $V_{\mathrm{m}}^{\mathrm{E}}$ are 0.314, 0.372, 0.230 and 0.303 cm^3^⋅mol^−1^, at mole fractions 0.205, 0.238, 0.217 and 0.175 (at *T*=348.15 K) for [BMIM][SCN], [BMPy][SCN], [BMPYR][SCN] and [BMPIP][SCN], respectively. At higher temperatures, the maximum of $V_{\mathrm{m}}^{\mathrm{E}}$ shifts to higher values of $V_{\mathrm{m}}^{\mathrm{E}}$ and to higher IL mole fraction. The absolute values of the maximum of the $V_{\mathrm{m}}^{\mathrm{E}}$ increase in the following order: [BMPYR][SCN] < [BMPIP][SCN] < [BMIM][SCN] < [BMPy][SCN]. This means that in the series of ILs, the strength of IL–water interactions is in agreement with the decreasing order observed. The lower positive $V_{\mathrm{m}}^{\mathrm{E}}$ values indicate the highest interaction with water and also that a more efficient packing and/or attractive interaction occurred. The negative deviations from ideality, observed for these systems, have to be the results of higher intermolecular interactions between the IL and water (electrostatic, dipole and hydrogen bonding) at the higher mole fraction of IL and at lower temperatures.

In recent papers [12–14] we reported the excess molar volumes for the mixtures of [BMIM][SCN] with different alcohols. The $V_{\mathrm{m}}^{\mathrm{E}}$ for mixtures of similar ILs with alcohols were negative. The polar interactions with alcohols are much stronger than with water.

It is well known that at low concentrations of water and, we assume, of the IL, strong association of water or of the IL, through hydrogen bonding, is expected. From the shape of the $V_{\mathrm{m}}^{\mathrm{E}}$ curves it can be assumed that cation/anion interactions of the IL are much weaker than hydrogen bonding in water. The negative values of $V_{\mathrm{m}}^{\mathrm{E}}$ at the IL-rich composition range underline this conclusion.

For the given system and for chosen number of parameters *A*_r_, the partial molar volumes $V_{1}^{\mathrm{E}}$ and $V_{2}^{\mathrm{E}}$ may be calculated using Eqs. 4–6: 4
5
6 Tables 8, 9, 10, and 11 show the values of the excess partial molar volumes, $V_{1}^{\mathrm{E}}$ and $V_{2}^{\mathrm{E}}$, calculated from the Redlich–Kister equation (Eqs. 2 and 3) at all experimental temperatures for the ILs (1) with water (2). In the system of [BMIM][SCN] with water the excess partial molar volumes, $V_{1}^{\mathrm{E}}$ of ionic liquid and $V_{2}^{\mathrm{E}}$ of water, showed S-shaped lines: partly negative values and partly positive values. The most positive values of $V_{2}^{\mathrm{E}}$ are for [BMPy][SCN], for dilute solutions of the ionic liquid. The positive effect of the disruption of the H-bond structure at high dilution of water, or an IL, and the negative effect in more concentrated solutions were observed as a result of partial H-bonds disruption, as was found by us previously for alcohols [12–14].

**Table 8:** The excess partial molar volumes, $V_{1}^{\mathrm{E}}$ and $V_{2}^{\mathrm{E}}$ for {[BMIM][SCN] (1) + water (2)} at different temperatures calculated using the Redlich–Kister equation

*x*_1_	${V_{1}^{\mathrm{E}}}\allowbreak (\mathrm{cm}^{3}{\cdot}\mathrm{mol}^{ - 1})$	${V_{2}^{\mathrm{E}}}\allowbreak (\mathrm{cm}^{3}{\cdot}\mathrm{mol}^{ - 1})$	*x*_1_	${V_{1}^{\mathrm{E}}}\allowbreak (\mathrm{cm}^{3}{\cdot}\mathrm{mol}^{ - 1})$	${V_{2}^{\mathrm{E}}}\allowbreak (\mathrm{cm}^{3}{\cdot}\mathrm{mol}^{ - 1})$
*T*=298.15 K					
0.9002	0.008	−0.221	0.3574	−0.177	0.021
0.7993	0.004	−0.204	0.2799	−0.248	0.054
0.6831	−0.021	−0.135	0.2051	−0.285	0.066
0.5654	−0.054	−0.080	0.1267	−0.190	0.050
0.4704	−0.094	−0.037	0.0556	0.170	0.016
*T*=308.15 K					
0.9002	0.013	−0.126	0.3574	−0.153	0.112
0.7993	0.009	−0.112	0.2799	−0.203	0.136
0.6831	−0.022	−0.024	0.2051	−0.188	0.132
0.5654	−0.054	0.031	0.1267	0.048	0.088
0.4704	−0.086	0.065	0.0556	0.698	0.027
*T*=318.15 K					
0.9002	0.014	−0.042	0.3574	−0.143	0.202
0.7993	0.008	−0.020	0.2799	−0.169	0.215
0.6831	−0.025	0.077	0.2051	−0.095	0.193
0.5654	−0.058	0.132	0.1267	0.282	0.122
0.4704	−0.087	0.163	0.0556	1.189	0.036
*T*=328.15 K					
0.9002	0.011	0.047	0.3574	−0.137	0.288
0.7993	0.003	0.085	0.2799	−0.144	0.292
0.6831	−0.028	0.173	0.2051	−0.014	0.252
0.5654	−0.055	0.220	0.1267	0.502	0.155
0.4704	−0.084	0.250	0.0556	1.657	0.044
*T*=338.15 K					
0.9002	0.018	0.107	0.3574	−0.1163	0.361
0.7993	0.010	0.140	0.2799	−0.1011	0.355
0.6831	−0.032	0.262	0.2051	0.0715	0.302
0.5654	−0.064	0.317	0.1267	0.694	0.184
0.4704	−0.084	0.338	0.0556	2.068	0.053
*T*=348.15 K					
0.9002	0.035	0.080	0.3574	−0.072	0.415
0.7993	0.012	0.181	0.2799	−0.113	0.434
0.6831	−0.063	0.403	0.2051	0.004	0.400
0.5654	−0.075	0.429	0.1267	0.737	0.263
0.4704	−0.047	0.400	0.0556	2.671	0.080

**Table 9:** The excess partial molar volumes, $V_{1}^{\mathrm{E}}$ and $V_{2}^{\mathrm{E}}$ for {[BMPy][SCN] (1) + water (2)} at different temperatures calculated using the Redlich−Kister equation

*x*_1_	${V_{1}^{\mathrm{E}}}\allowbreak (\mathrm{cm}^{3}{\cdot}\mathrm{mol}^{ - 1})$	${V_{2}^{\mathrm{E}}}\allowbreak (\mathrm{cm}^{3}{\cdot}\mathrm{mol}^{ - 1})$	*x*_1_	${V_{1}^{\mathrm{E}}}\allowbreak (\mathrm{cm}^{3}{\cdot}\mathrm{mol}^{ - 1})$	${V_{2}^{\mathrm{E}}}\allowbreak (\mathrm{cm}^{3}{\cdot}\mathrm{mol}^{ - 1})$
*T*=298.15 K					
0.9436	0.005	−0.242	0.3001	−0.233	0.063
0.8345	0.001	−0.239	0.2377	−0.293	0.086
0.7220	−0.033	−0.120	0.1667	−0.284	0.085
0.6203	−0.061	−0.060	0.0972	−0.045	0.051
0.5372	−0.078	−0.037	0.0430	0.458	0.015
0.4331	−0.114	−0.004	0.0286	0.662	0.007
0.3636	−0.166	0.030	0.0076	1.026	0.001
*T*=308.15 K					
0.9436	0.006	−0.120	0.3001	−0.190	0.160
0.8345	0.001	−0.112	0.2377	−0.219	0.172
0.7220	−0.037	0.021	0.1667	−0.116	0.147
0.6203	−0.061	0.073	0.0972	0.338	0.082
0.5372	−0.069	0.084	0.0430	1.158	0.023
0.4331	−0.092	0.105	0.0286	1.478	0.011
0.3636	−0.136	0.134	0.0076	2.040	0.001
*T*=318.15 K					
0.9436	0.006	−0.025	0.3001	−0.153	0.251
0.8345	−0.001	−0.007	0.2377	−0.146	0.250
0.7220	−0.043	0.143	0.1667	0.050	0.203
0.6203	−0.067	0.196	0.0972	0.697	0.109
0.5372	−0.069	0.200	0.0430	1.789	0.030
0.4331	−0.083	0.211	0.0286	2.206	0.014
0.3636	−0.116	0.233	0.0076	2.932	0.001
*T*=328.15 K					
0.9436	0.021	0.069	0.3001	−0.145	0.335
0.8345	0.019	−0.024	0.2377	−0.238	0.371
0.7220	−0.074	0.308	0.1667	−0.076	0.334
0.6203	−0.104	0.379	0.0972	0.903	0.194
0.5372	−0.057	0.315	0.0430	2.825	0.055
0.4331	0.001	0.258	0.0286	3.595	0.027
0.3636	−0.040	0.284	0.0076	4.966	0.002
*T*=338.15 K					
0.9436	0.014	0.183	0.3001	−0.092	0.419
0.8345	0.012	0.129	0.2377	−0.064	0.410
0.7220	−0.057	0.375	0.1667	0.259	0.332
0.6203	−0.089	0.445	0.0972	1.314	0.179
0.5372	−0.069	0.419	0.0430	3.110	0.049
0.4331	−0.041	0.391	0.0286	3.798	0.024
0.3636	−0.059	0.403	0.0076	5.003	0.002
*T*=348.15 K					
0.9436	0.010	0.282	0.3001	−0.059	0.506
0.8345	0.004	0.272	0.2377	0.049	0.467
0.7220	−0.049	0.463	0.1667	0.504	0.357
0.6203	−0.076	0.523	0.0972	1.690	0.183
0.5372	−0.070	0.514	0.0430	3.540	0.049
0.4331	−0.060	0.505	0.0286	4.227	0.023
0.3636	−0.069	0.510	0.0076	5.412	0.002

**Table 10:** The excess partial molar volumes, $V_{1}^{\mathrm{E}}$ and $V_{2}^{\mathrm{E}}$ for {[BMPYR][SCN] (1) + water (2)} at different temperatures calculated using the Redlich–Kister equation

*x*_1_	${V_{1}^{\mathrm{E}}}\allowbreak (\mathrm{cm}^{3}{\cdot}\mathrm{mol}^{ - 1})$	${V_{2}^{\mathrm{E}}}\allowbreak (\mathrm{cm}^{3}{\cdot}\mathrm{mol}^{ - 1})$	*x*_1_	${V_{1}^{\mathrm{E}}}\allowbreak (\mathrm{cm}^{3}{\cdot}\mathrm{mol}^{ - 1})$	${V_{2}^{\mathrm{E}}}\allowbreak (\mathrm{cm}^{3}{\cdot}\mathrm{mol}^{ - 1})$
*T*=298.15 K					
0.9147	−0.007	−0.688	0.3366	−0.452	−0.040
0.7797	−0.048	−0.454	0.2170	−0.728	0.067
0.6445	−0.094	−0.338	0.1377	−0.731	0.071
0.4748	−0.201	−0.208	0.0343	0.047	0.010
*T*=308.15 K					
0.9147	0.016	−0.576	0.3366	−0.378	0.042
0.7797	−0.026	−0.387	0.2170	−0.653	0.150
0.6445	−0.107	−0.176	0.1377	−0.563	0.135
0.4748	−0.174	−0.091	0.0343	0.906	0.019
*T*=318.15 K					
0.9147	0.016	−0.429	0.3366	−0.363	0.140
0.7797	−0.019	−0.274	0.2170	−0.556	0.218
0.6445	−0.094	−0.081	0.1377	−0.320	0.173
0.4748	−0.167	0.010	0.0343	1.539	0.022
*T*=328.15 K					
0.9147	0.013	−0.354	0.3366	−0.346	0.230
0.7797	−0.026	−0.169	0.2170	−0.454	0.277
0.6445	−0.101	0.025	0.1377	−0.087	0.204
0.4748	−0.172	0.115	0.0343	2.098	0.025
*T*=338.15 K					
0.9147	0.011	−0.238	0.3366	−0.284	0.290
0.7797	−0.019	−0.103	0.2170	−0.202	0.265
0.6445	−0.100	0.103	0.1377	0.240	0.174
0.4748	−0.198	0.230	0.0343	2.085	0.019
*T*=348.15 K					
0.9147	0.010	−0.115	0.3366	−0.274	0.382
0.7797	−0.013	−0.015	0.2170	−0.074	0.314
0.6445	−0.093	0.182	0.1377	0.505	0.194
0.4748	−0.211	0.335	0.0343	2.544	0.020

**Table 11:** The excess partial molar volumes, $V_{1}^{\mathrm{E}}$ and $V_{2}^{\mathrm{E}}$ for {[BMPIP][SCN] (1) + water (2)} at different temperatures calculated using the Redlich–Kister equation

*x*_1_	${V_{1}^{\mathrm{E}}}\allowbreak (\mathrm{cm}^{3}{\cdot}\mathrm{mol}^{ - 1})$	${V_{2}^{\mathrm{E}}}\allowbreak (\mathrm{cm}^{3}{\cdot}\mathrm{mol}^{ - 1})$	*x*_1_	${V_{1}^{\mathrm{E}}}\allowbreak (\mathrm{cm}^{3}{\cdot}\mathrm{mol}^{ - 1})$	${V_{2}^{\mathrm{E}}}\allowbreak (\mathrm{cm}^{3}{\cdot}\mathrm{mol}^{ - 1})$
*T*=318.15 K					
0.8954	0.002	−0.403	0.3698	−0.251	0.115
0.7226	−0.078	−0.058	0.2828	−0.430	0.202
0.6259	−0.106	0.003	0.1752	−0.459	0.218
0.4889	−0.124	0.023	0.0649	0.817	0.069
*T*=328.15 K					
0.8954	0.012	−0.396	0.3698	−0.278	0.222
0.7226	−0.083	−0.010	0.2828	−0.414	0.288
0.6259	−0.133	0.098	0.1752	−0.319	0.269
0.4889	−0.169	0.143	0.0649	1.271	0.080
*T*=338.15 K					
0.8954	−0.003	−0.209	0.3698	−0.237	0.322
0.7226	−0.085	0.145	0.2828	−0.302	0.355
0.6259	−0.120	0.220	0.1752	−0.045	0.288
0.4889	−0.152	0.259	0.0649	1.678	0.079
*T*=348.15 K					
0.8954	−0.009	−0.021	0.3698	−0.240	0.441
0.7226	−0.066	0.230	0.2828	−0.215	0.431
0.6259	−0.100	0.303	0.1752	0.251	0.303
0.4889	−0.166	0.384	0.0649	2.095	0.074

**Fig. 6:**
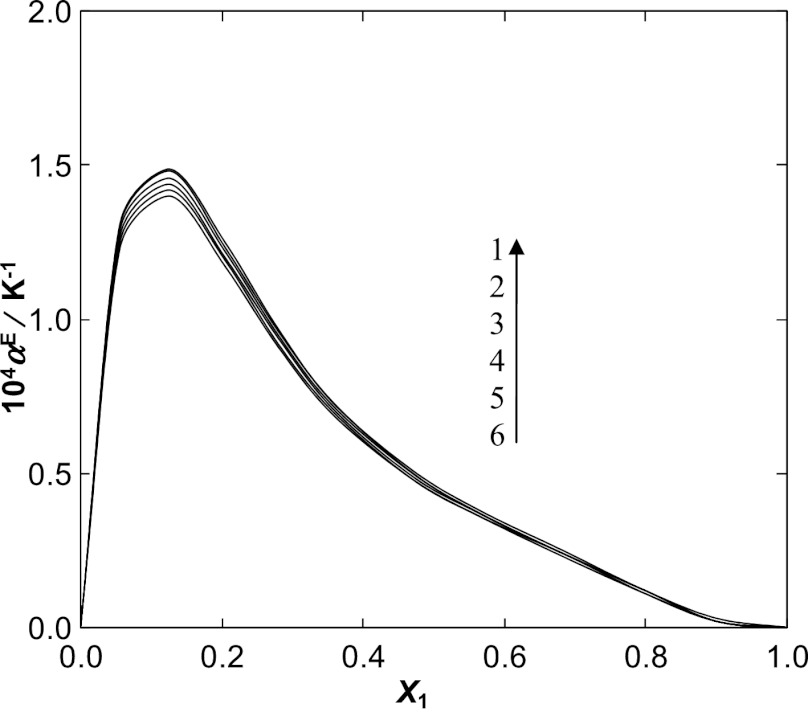
Plot of the excess isobaric expansivity *α* ^E^ of the {[BMIM][SCN] (1) + water (2)} binary system against mole fraction *x*_1_ at different temperatures: (**1**) 298.15 K, (**2**) 308.15 K, (**3**) 318.15 K, (**4**) 328.15 K, (**5**) 338.15 K, (**6**) 348.15 K

The deviation in the viscosity, Δ*η*, were obtained from the relation:

7 $$ \Delta\eta = \eta - (x_{1}\eta_{1} + x_{2}\eta_{2}) $$

where *η* is the absolute dynamic viscosity of the mixture, and *η*_1_ and *η*_2_ are the viscosities of the pure components. The values of Δ*η* of the binary mixtures are presented in Tables 3, 4, 5, and 6 and were fitted to a Redlich–Kister polynomial equation by the method of least squares, in order to derive the binary solution coefficients. See Table 8S in the ESM. Figure 4 depicts the deviation in the viscosity as a function of the IL mole fraction at different temperatures for [BMPy][SCN] as an example. The rest of the data are shown in Fig. 6S a–c in the ESM.

From the temperature dependence of density, the isobaric expansivity (coefficient of thermal expansion), *α*, defined by Eq. 1, can be calculated (*α*=5.55×10^−4^ K^−1^, *α*=5.40×10^−4^ K^−1^, *α*=5.08×10^−4^ K^−1^, and *α*=5.50×10^−4^ K^−1^, at *T*=298.15 K for [BMIM][SCN], [BMPy][SCN], [BMPYR][SCN] and [BMPIP][SCN], respectively). 8 The values of *α* are listed in Tables 3, 4, 5, and 6. The character of changes of *α* as a function of mole fraction of the IL for different temperatures is shown in Fig. 5 as an example for [BMIM][SCN]. The results for the other three ILs are presented in Figs. 7 a–c in the ESM. This function exhibits maxima at low concentration of the IL, which is different from the mixtures of [BMIM][SCN] with alcohols [12–14]. The values of *α* increases with an increase of temperature, which is typical.

Additionally, the corresponding excess function was determined for all binary systems. The excess isobaric expansivity was calculated using the equation:

9 $$ \alpha^{\mathrm{E}} = \alpha - \varphi_{1}^{\mathrm{id}} \alpha_{1} - \varphi_{2}^{\mathrm{id}}\alpha_{2} $$

where $\varphi_{i}^{\mathrm{id}}$ is an ideal volume fraction given by the following relation:

10 $$ \varphi_{1}^{\mathrm{id}} = \frac{x_{i}V_{\mathrm{m}i}}{x_{1}V_{\mathrm{m}1} + x_{2}V_{\mathrm{m}2}} $$

in which *V*_m*i*_ stands for the molar volume for a pure component *i*. For these calculations it was necessary to determine the temperature dependence of the Redlich–Kister parameters, described earlier (Eqs. 3 and 4). An increasing excess isobaric expansivity with deceasing temperature is observed as is shown in Fig. 6 and in Figs. 8S a–c in the ESM. The curves are asymmetrical, with the maxima of *α* ^E^ located at low mole fraction of the IL and decreasing values of *α* ^E^ with increasing IL concentration (see Fig. 6 for [BMIM][SCN] and Figs. 8S a–c in the ESM). This is connected with the $V_{\mathrm{m}}^{\mathrm{E}}$ function and interactions in the solution.

## Conclusions

In this paper, new data on densities and viscosities of four pure ionic liquids, [BMIM][SCN], [BMPy][SCN], [BMPYR][SCN], and [BMPIP][SCN] and their mixtures with water have been presented. S-shape deviations were observed for excess molar volumes with a maximum at water-rich composition and at high temperatures, which is mainly a result of the disruption of the hydrogen bonded associates of the water molecules. Small minima were observed in the IL-rich composition, which is the result of the interaction between the ILs and water. The deviations in the viscosity were negative for all binary systems. Any changes in the volumetric behavior of the mixtures can be related to changes in a single molecular entity, i.e., the isobaric expansivity, *α*, and the excess isobaric expansivity, *α* ^E^, which for the ILs investigated here have maxima at the same range of composition. These properties are different from that observed earlier for the system of [BMIM][SCN] with alcohols.

The results of the correlations with the second order polynomials, Redlich–Kister equation, and VFT equation of density, viscosity, excess molar volume, and viscosity deviation are with very low standard deviations.

## Electronic Supplementary Material

Below is the link to the electronic supplementary material.

Electronic Supplementary Material available: DSC and TG/DTA for [BMPy][SCN] (GRS 1, Fig. 1S); fit parameters and standard deviation for the empirical correlation of density in the systems {IL (1) + water (2)} (Tables 1S, 2S and Fig 2S); fit parameters of the VFT equation and the relative standard deviations for the correlation of viscosity as a function of temperature in the systems {[IL] (1) + water (2)} (Tables 3S, 4S, and Fig. 3S); coefficients of polynomial for the correlation of density and viscosity in function of IL mole fraction in the systems {IL (1) + water (2)} (Tables 5S, 6S and Figs. 4S, 5S); coefficients of the Redlich–Kister equation for the correlation of the deviation in viscosity along with the root-mean-square deviation, (Table 7S and Fig. 6S); plot of isobaric expansivities and excess isobaric expansivities as a function of concentration (Figs. 7, 8). This material is available free of charge via the Internet at http:// Journal of Solution Chemistry (DOC 1.1 MB)
