# Supplementary material for: Density and Viscosity of Binary Mixtures of Thiocyanate Ionic Liquids + Water as a Function of Temperature
Source: J Solution Chem. 2012 Aug 21;41(8):1422–45. doi: 10.1007/s10953-012-9875-7 (PMC3434890; doi:10.1007/s10953-012-9875-7)
Supplement: Supplementary file 1 — Electronic Supplementary Material available: DSC and TG/DTA for [BMPy][SCN] (GRS 1, Fig. 1S); fit parameters and standard deviation for the empirical correlation of density in the systems {IL (1) + water (2)} (Tables 1S, 2S and Fig 2S); fit parameters of the VFT equation and the relative standard deviations for the correlation of viscosity as a function of temperature in the systems {[IL] (1) + water (2)} (Tables 3S, 4S, and Fig. 3S); coefficients of polynomial for the correlation of density and viscosity in function of IL mole fraction in the systems {IL (1) + water (2)} (Tables 5S, 6S and Figs. 4S, 5S); coefficients of the Redlich–Kister equation for the correlation of the deviation in viscosity along with the root-mean-square deviation, (Table 7S and Fig. 6S); plot of isobaric expansivities and excess isobaric expansivities as a function of concentration (Figs. 7, 8). This material is available free of charge via the Internet at http:// Journal of Solution Chemistry (DOC 1.1 MB) [file 10953_2012_9875_MOESM1_ESM.doc]

**Journal of Solution Chemistry**

Supplementary Material

Density and Viscosity of Binary Mixtures of Thiocyanate Ionic Liquids + Water as a Function of Temperature

U. Domańska ⋅ M. Królikowska

U. Domańska (🖂) ⋅ M. Królikowska

Department of Physical Chemistry, Faculty of Chemistry, Warsaw University of Technology, Noakowskiego 3, 00-664 Warsaw, Poland,

e-mail: [ula@ch.pw.edu.pl](mailto:ula@ch.pw.edu.pl).

U. Domańska

Thermodynamic Research Unit, School of Chemical Engineering, University of KwaZulu-Natal, Howard College Campus, King George V Avenue, Durban 4001, South Africa

**Table 1S** Fit parameters $a_{0}$, $a_{1}$ and $a_{2}$with $R^{2}=1$ for empirical correlation^a^ of the density as a function of temperature for pure ILs

|  | $10^{7}\cdot a_{2}/$ $\text{g}\cdot\text{cm}^{-3}\cdot\text{K}^{-2}$ | $10^{4}\cdot a_{1}/$ $\text{g}\cdot\text{cm}^{-3}\cdot\text{K}^{-1}$ | $a_{0}/$ $\text{g}\cdot\text{cm}^{-3}$ |
| --- | --- | --- | --- |
| [BMIM][SCN] | 2.30 | −7.31 | 1.2672 |
| [BMPy][SCN] | 1.64 | −6.70 | 1.2464 |
| [BMPYR][SCN] | 0.63 | −5.58 | 1.1854 |
| [BMPIP][SCN] | 6.50 | −9.48 | 1.2555 |

^a^ Equation used:

$$\rho/\text{g}\cdot\text{cm}^{-3}=a_{0}+a_{1}(T/K)+a_{2}(T/K)^{2}$$

**Table 2S** Fit Parameters $a_{2}$, $a_{1}$, $a_{0}$ and $R^{2}$ for the empirical correlation^a^ of density of temperature for mixtures {IL (1) + water (2)}

|  |  |  |  |
| --- | --- | --- | --- |
| *x*_1_ | $10^{7}\cdot a_{2}/$ $\text{g}\cdot\text{cm}^{-3}\cdot\text{K}^{-2}$ | $10^{4}\cdot a_{1}/$ $\text{g}\cdot\text{cm}^{-3}\cdot\text{K}^{-1}$ | $a_{0}/$ $\text{g}\cdot\text{cm}^{-3}$ |
| [BMIM][SCN] (1) + water (2) | | | |
| 1.0000 | 2.30 | −7.31 | 1.2672 |
| 0.9002 | 2.10 | −7.24 | 1.2660 |
| 0.7993 | 2.10 | −7.29 | 1.2666 |
| 0.6831 | 1.66 | −7.06 | 1.2627 |
| 0.5654 | 1.21 | −6.87 | 1.2595 |
| 0.4704 | 0.66 | −6.63 | 1.2551 |
| 0.3574 | 0.36 | −6.15 | 1.2460 |
| 0.2799 | 1.75 | −5.40 | 1.2320 |
| 0.2051 | −3.36 | −4.48 | 1.2135 |
| 0.1267 | −6.88 | −2.21 | 1.1671 |
| 0.0556 | −4.13 | −3.64 | 1.1695 |
| 0.0000 | −33.82 | 17.37 | 0.7799 |
| [BMPy][SCN] (1) + water (2) | | | |
| 1.0000 | 1.64 | −6.70 | 1.2464 |
| 0.9436 | 1.55 | −6.67 | 1.2460 |
| 0.8345 | 1.43 | −6.65 | 1.2459 |
| 0.7220 | 1.34 | −6.67 | 1.2465 |
| 0.6203 | 1.16 | −6.65 | 1.2463 |
| 0.5372 | 0.75 | −6.49 | 1.2439 |
| 0.4331 | 0.32 | −6.37 | 1.2421 |
| 0.3636 | −0.23 | −6.15 | 1.2382 |
| 0.3001 | −1.09 | −5.74 | 1.2309 |
| 0.2377 | −2.41 | −5.03 | 1.2179 |
| 0.1667 | −4.36 | −3.89 | 1.1954 |
| 0.0972 | 5.36 | −3.18 | 1.1732 |
| 0.0430 | 1.20 | −6.89 | 1.2117 |
| 0.0286 | 9.45 | 0.00 | 1.0955 |
| 0.0076 | 1.38 | −6.09 | 1.1707 |
| 0.0000 | −33.82 | 17.37 | 0.7799 |
| [BMPYR][SCN] (1) + water (2) | | | |
| 1.0000 | 0.63 | −5.58 | 1.1854 |
| 0.9147 | 1.30 | −6.08 | 1.1944 |
| 0.7797 | 1.14 | −6.05 | 1.1949 |
| 0.6445 | 0.91 | −6.01 | 1.1958 |
| 0.4748 | 0.46 | −5.97 | 1.1973 |
| 0.3366 | −0.57 | −5.56 | 1.1936 |
| 0.2170 | −0.95 | −5.55 | 1.1936 |
| 0.1377 | 0.82 | −6.63 | 1.2058 |
| 0.0343 | −3.27 | −3.32 | 1.1321 |
| 0.0000 | −33.82 | 17.37 | 0.7799 |
| [BMPIP][SCN] (1) + water (2) | | | |
| 1.0000 | 6.50 | −9.48 | 1.2555 |
| 0.8954 | 5.25 | −8.71 | 1.2434 |
| 0.7226 | 2.00 | −6.66 | 1.2107 |
| 0.6259 | −0.25 | −5.30 | 1.1899 |
| 0.4889 | 0.00 | −5.66 | 1.1978 |
| 0.3698 | −1.00 | −5.24 | 1.1929 |
| 0.2828 | −1.50 | −5.15 | 1.1930 |
| 0.1752 | −4.25 | −3.63 | 1.1677 |
| 0.0649 | 8.00 | −11.23 | 1.2755 |
| 0.0000 | −26.00 | 12.15 | 0.8669 |

^a^ Parameters from the equation: $\rho/\text{g}\cdot\text{cm}^{-3}=a_{0}+a_{1}(T/K)+a_{2}(T/K)^{2}$

**Table 3S** Fit parameters of the VFT equation for the correlation of viscosity as a function of temperature in binary systems of {IL (1) + water (2)}

| $x_{1}$ | $\frac{10^{-3}A}{\text{mPa}\cdot\text{s}\cdot\text{K}^{-0,5}}$ | $\frac{10^{3}B}{K}$ |
| --- | --- | --- |
| [BMIM][SCN] (1) + water (2) | | |
| 1.0000 | 1.32 | 1.34 |
| 0.9002 | 1.21 | 1.91 |
| 0.7993 | 1.17 | 2.07 |
| 0.6831 | 1.11 | 2.20 |
| 0.5654 | 1.07 | 2.22 |
| 0.4704 | 1.04 | 2.06 |
| 0.3574 | 1.01 | 1.75 |
| 0.2799 | 0.98 | 1.58 |
| 0.2051 | 0.93 | 1.44 |
| 0.1267 | 0.87 | 1.33 |
| 0.0556 | 0.78 | 1.21 |
| 0.0000 | 0.56 | 1.93 |
| [BMPy][SCN] (1) + water (2) | | |
| 1.0000 | 1.23 | 1.62 |
| 0.9436 | 1.20 | 1.70 |
| 0.8345 | 1.14 | 1.88 |
| 0.7220 | 1.08 | 2.10 |
| 0.6203 | 1.01 | 2.39 |
| 0.5372 | 0.96 | 2.74 |
| 0.4331 | 0.91 | 2.72 |
| 0.3636 | 0.88 | 2.63 |
| 0.2377 | 0.84 | 2.10 |
| 0.1667 | 0.86 | 1.32 |
| 0.0972 | 0.73 | 1.79 |
| 0.0430 | 0.65 | 1.59 |
| 0.0286 | 0.61 | 1.68 |
| 0.0076 | 0.50 | 2.37 |
| 0.0000 | 0.46 | 2.52 |
| [BMPYR][SCN] (1) + water (2) | | |
| 1.0000 | 1.40 | 1.95 |
| 0.9147 | 1.39 | 1.75 |
| 0.7797 | 1.32 | 1.89 |
| 0.6445 | 1.28 | 1.79 |
| 0.4748 | 1.17 | 1.87 |
| 0.3366 | 1.12 | 1.55 |
| 0.2170 | 1.09 | 1.07 |
| 0.1377 | 1.04 | 0.85 |
| 0.0343 | 0.77 | 1.14 |
| 0.0000 | 0.57 | 1.87 |
| [BMPIP][SCN] (1) + water (2) | | |
| 1.0000 | 1.76 | 0.53 |
| 0.7226 | 1.48 | 1.00 |
| 0.6259 | 1.36 | 1.16 |
| 0.4889 | 1.23 | 1.42 |
| 0.3698 | 1.12 | 1.57 |
| 0.2828 | 1.06 | 1.44 |
| 0.1752 | 0.98 | 1.13 |
| 0.0649 | 0.76 | 1.36 |
| 0.0000 | 0.49 | 2.10 |

**Table 4S** The values of the root-mean-square deviations for the correlation of viscosity dependence on temperature with VFT equation, $\sigma$a/ mPa∙s for binary systems of {IL (1) + water (2)}

|  | *T* / K | | | | | |
| --- | --- | --- | --- | --- | --- | --- |
|  | 298.15 | 308.15 | 318.15 | 328.15 | 338.15 | 348.15 |
| [BMIM][SCN] (1) + water (2) | 0.31 | 0.16 | 0.16 | 0.18 | 0.03 | 0.08 |
| [BMPy][SCN] (1) + water (2) | 0.62 | 0.21 | 0.21 | 0.09 | 0.02 | 0.09 |
| [BMPYR][SCN] (1) + water (2) | 0.70 | 0.19 | 0.32 | 0.15 | 0.02 | 0.11 |
| [BMPIP][SCN] (1) + water (2) |  |  | 0.46 | 0.43 | 0.12 | 0.10 |

^a^ Root-mean square deviation $\sigma=\sqrt{\sum_{i=1}^{n} \frac{(\eta_{\exp}-\eta_{calc})^{2}}{(n-2)}}$

**Table 5S** Coefficients of polynomial^a^ for the correlation of the density, *ρ*/g·cm^−3^ as a function of concentration at different temperatures of the binary systems {IL (1) + water (2)}

|  | $c_{6}$ | $c_{5}$ | $c_{4}$ | $c_{3}$ | $c_{2}$ | $c_{1}$ | $c_{0}$ |
| --- | --- | --- | --- | --- | --- | --- | --- |
| [BMIM][SCN] (1) + water (2) | | | | | | | |
| 298.15 | −2.269 | 7.781 | −10.562 | 7.279 | −2.750 | 0.594 | 0.9975 |
| 308.15 | −1.587 | 5.547 | −7.732 | 5.535 | −2.213 | 0.520 | 0.9948 |
| 318.15 | −1.296 | 4.558 | −6.410 | 4.656 | −1.912 | 0.472 | 0.9904 |
| 328.15 | −1.048 | 3.709 | −5.269 | 3.897 | −1.654 | 0.432 | 0.9858 |
| 338.15 | −0.852 | 3.049 | −4.399 | 3.327 | −1.463 | 0.404 | 0.9804 |
| 348.15 | −0.740 | 2.648 | −3.830 | 2.922 | −1.314 | 0.380 | 0.9748 |
| [BMPy][SCN] (1) + water (2) | | | | | | | |
| 298.15 |  | 0.921 | −2.725 | 3.072 | −1.668 | 0.464 | 0.998 |
| 308.15 |  | 0.761 | −2.271 | 2.590 | −1.437 | 0.417 | 0.995 |
| 318.15 |  | 0.617 | −1.860 | 2.157 | −1.232 | 0.377 | 0.991 |
| 328.15 |  | 0.656 | −1.975 | 2.286 | −1.300 | 0.396 | 0.982 |
| 338.15 |  | 0.583 | −1.751 | 2.029 | −1.166 | 0.367 | 0.978 |
| 348.15 |  | 3.334 | −1.042 | 1.284 | −0.815 | 0.298 | 0.975 |
| [BMPYR][SCN] (1) + water (2) | | | | | | | |
| 298.15 |  | 0.300 | −0.931 | 1.124 | −0.670 | 0.205 | 0.997 |
| 308.15 |  | 0.142 | −0.483 | 0.654 | −0.449 | 0.162 | 0.994 |
| 318.15 |  | 0.031 | −0.162 | 0.309 | −0.282 | 0.129 | 0.990 |
| 328.15 |  | −0.052 | 0.080 | 0.046 | −0.153 | 0.103 | 0.985 |
| 338.15 |  | 0.003 | −0.058 | 0.160 | −0.185 | 0.103 | 0.980 |
| 348.15 |  | −0.037 | 0.070 | 0.010 | −0.107 | 0.088 | 0.974 |
| [BMPIP][SCN] (1) + water (2) | | | | | | | |
| 318.15 |  |  | −0.131 | 0.351 | −0.348 | 0.157 | 0.990 |
| 328.15 |  |  | −0.087 | 0.250 | −0.271 | 0.137 | 0.985 |
| 338.15 |  |  | −0.069 | 0.205 | −0.234 | 0.126 | 0.980 |
| 348.15 |  |  | −0.048 | 0.156 | −0.194 | 0.116 | 0.974 |

^a^ Polynomial:$\rho=c_{6}\cdot x_{1}^{6}+c_{5}\cdot x_{1}^{5}+c_{4}\cdot x_{1}^{4}+c_{3}\cdot x_{1}^{3}+c_{2}\cdot x_{1}^{2}+c_{1}\cdot x_{1}+c_{0}$

**Table 6S** Coefficients of polynomial^a^ for the correlation of the viscosity, *η* /mPa·s as a function of concentration at different temperatures of the binary systems {IL (1) + water (2)}

|  | $d_{4}$ | $d_{3}$ | $d_{2}$ | $d_{1}$ | $d_{0}$ |
| --- | --- | --- | --- | --- | --- |
| [BMIM][SCN] (1) + water (2) | | | | | |
| 298.15 | 85.173 | −132.010 | 87.702 | 9.589 | 1.086 |
| 308.15 | 44.711 | −71.192 | 50.677 | 9.215 | 0.858 |
| 318.15 | 23.866 | −39.690 | 30.732 | 8.192 | 0.707 |
| 328.15 | 12.704 | −22.676 | 19.467 | 7.072 | 0.601 |
| 338.15 | 6.549 | −13.176 | 12.845 | 6.042 | 0.522 |
| 348.15 | 3.086 | −7.732 | 8.815 | 5.152 | 0.462 |
| [BMPy][SCN] (1) + water (2) | | | | | |
| 298.15 | −14.226 | 85.947 | −19.543 | 32.700 | 0.737 |
| 308.15 | −18.869 | 63.144 | −17.640 | 24.440 | 0.613 |
| 318.15 | −11.354 | 35.667 | −8.736 | 17.760 | 0.545 |
| 328.15 | −3.433 | 14.198 | −0.491 | 12.758 | 0.507 |
| 338.15 | 2.373 | −1.100 | 6.477 | 8.818 | 0.484 |
| 348.15 | 4.199 | −6.640 | 8.337 | 6.503 | 0.454 |
| [BMPYR][SCN] (1) + water (2) | | | | | |
| 298.15 | −10.091 | 42.706 | 46.217 | 29.624 | 0.818 |
| 308.15 | −0.422 | 10.889 | 38.483 | 20.533 | 0.707 |
| 318.15 | 8.085 | −12.791 | 37.876 | 13.760 | 0.632 |
| 328.15 | 10.574 | −20.129 | 32.996 | 9.944 | 0.568 |
| 338.15 | 12.464 | −24.830 | 29.926 | 7.120 | 0.514 |
| 348.15 | 13.805 | −27.477 | 27.196 | 5.285 | 0.460 |
| [BMPIP][SCN] (1) + water (2) | | | | | |
| 318.15 | 146.079 | 119.653 | −41.431 | 40.292 | 0.103 |
| 328.15 | 42.411 | 115.589 | −35.873 | 29.404 | 0.201 |
| 338.15 | 6.361 | 90.292 | −24.650 | 21.361 | 0.256 |
| 348.15 | −12.302 | 75.812 | −19.593 | 16.399 | 0.262 |

^a^ Polynomial $\eta=d_{4}\cdot x_{1}^{4}+d_{3}\cdot x_{1}^{3}+d_{2}\cdot x_{1}^{2}+d_{1}\cdot x_{1}+d_{0}$

**Table 7S** The values of the root-mean square deviations, $\sigma$a/ cm^3^∙mol^−1^ for the correlation of excess molar volume dependence on temperature with Redlich – Kister equation for binary systems of {IL (1) + water (2)}

|  | *T* / K | | | | | |
| --- | --- | --- | --- | --- | --- | --- |
|  | 298.15 | 308.15 | 318.15 | 328.15 | 338.15 | 348.15 |
| [BMIM][SCN] (1) + water (2) | 0.0027 | 0.0029 | 0.0035 | 0.0045 | 0.0039 | 0.0196 |
| [BMPy][SCN] (1) + water (2) | 0.0066 | 0.0118 | 0.0136 | 0.0317 | 0.0273 | 0.0182 |
| [BMPYR][SCN] (1) + water (2) | 0.0059 | 0.0088 | 0.0113 | 0.0106 | 0.0146 | 0.0093 |
| [BMPIP][SCN] (1) + water (2) |  |  | 0.0073 | 0.0076 | 0.0025 | 0.0081 |

^a^ Root-mean square deviation $\sigma=\sqrt{\sum_{i=1}^{n} \frac{(\rho_{\exp}-\rho_{calc})^{2}}{(n-2)}}$

**Table 8S** Coefficients of the Redlich –Kister equation^a^ for the correlation of the deviations in viscosity, *Δη* /mPa·s as a function of temperature and the root-mean square deviation^b^ for the binary systems {IL (1) + water (2)}

| *T* / (K) | $\frac{B_{1}}{\text{mPa}\cdot\text{s}}$ | $\frac{B_{2}}{\text{mPa}\cdot\text{s}}$ | $\frac{B_{3}}{\text{mPa}\cdot\text{s}}$ | $\frac{B_{4}}{\text{mPa}\cdot\text{s}}$ | $\frac{\sigma}{\text{mPa}\cdot\text{s}}$ |
| --- | --- | --- | --- | --- | --- |
| [BMIM][SCN] (1) + water (2) | | | | | |
| 298.15 | −38.44 | −11.31 | −16.61 | −18.97 | 0.29 |
| 308.15 | −23.05 | −6.712 | −15.17 | −16.92 | 0.24 |
| 318.15 | −13.47 | −2.891 | −10.34 | −11.31 | 0.12 |
| 328.15 | −6.699 | 0.7768 | −2.514 | −3.450 | 0.03 |
| 338.15 | −4.371 | 1.188 | −1.267 | −2.727 | 0.03 |
| 348.15 | −2.956 | 0.9516 | −0.5443 | −1.232 | 0.03 |
| [BMPy][SCN] (1) + water (2) | | | | | |
| 298.15 | −84.89 | −30.75 | 2.611 | 6.529 | 0.29 |
| 308.15 | −44.38 | −14.57 | 4.221 | 6.452 | 0.19 |
| 318.15 | −25.07 | −8.040 | 2.643 | 5.354 | 0.14 |
| 328.15 | −14.87 | −4.787 | 0.9598 | 4.053 | 0.08 |
| 338.15 | −8.979 | −2.434 | −0.3893 | 2.142 | 0.06 |
| 348.15 | −5.747 | −0.9911 | −1.125 | 0.2953 | 0.07 |
| [BMPYR][SCN] (1) + water (2) | | | | | |
| 298.15 | −92.98 | −8.074 | 0.7986 | −10.30 | 0.39 |
| 308.15 | −54.37 | −1.946 | −1.267 | −10.16 | 0.31 |
| 318.15 | −33.14 | 1.879 | −3.603 | −12.56 | 0.32 |
| 328.15 | −21.55 | 2.088 | −3.722 | −9.367 | 0.24 |
| 338.15 | −14.63 | 1.411 | −3.717 | −5.360 | 0.14 |
| 348.15 | −10.25 | 1.261 | −4.015 | −4.603 | 0.09 |
| [BMPIP][SCN] (1) + water (2) | | | | | |
| 318.15 | −398.9 | −230.4 | 15.50 | 135.9 | 1.93 |
| 328.15 | −215.2 | −116.2 | 23.49 | 89.21 | 1.16 |
| 338.15 | −124.3 | −62.62 | 21.96 | 61.49 | 0.77 |
| 348.15 | −74.26 | −33.30 | 19.43 | 42.61 | 0.55 |

^a^ According to eqn. $V_{\mathrm{m}}^{\mathrm{E}} = x_{1} (x_{1}- 1) \sum_{r = 0}^{r = 6} B_{\text{r}}(1 - 2x_{1})^{r-1}$

^b^ According to eqn. $\sigma\text{ }=\text{ }\left\{\sum_{i=1}^{n} \left(V_{\text{m(expt)}}^{\text{E}}\text{ - }V_{\text{m(calc)}}^{\text{E}}\right)_{\text{i}}^{2}/\left(n\text{ - }k\right)\right\}^{\text{1/2}}\text{ }$

**GRS. 1** The differential scanning calorimetry, DSC, diagram for [BMPy][SCN]


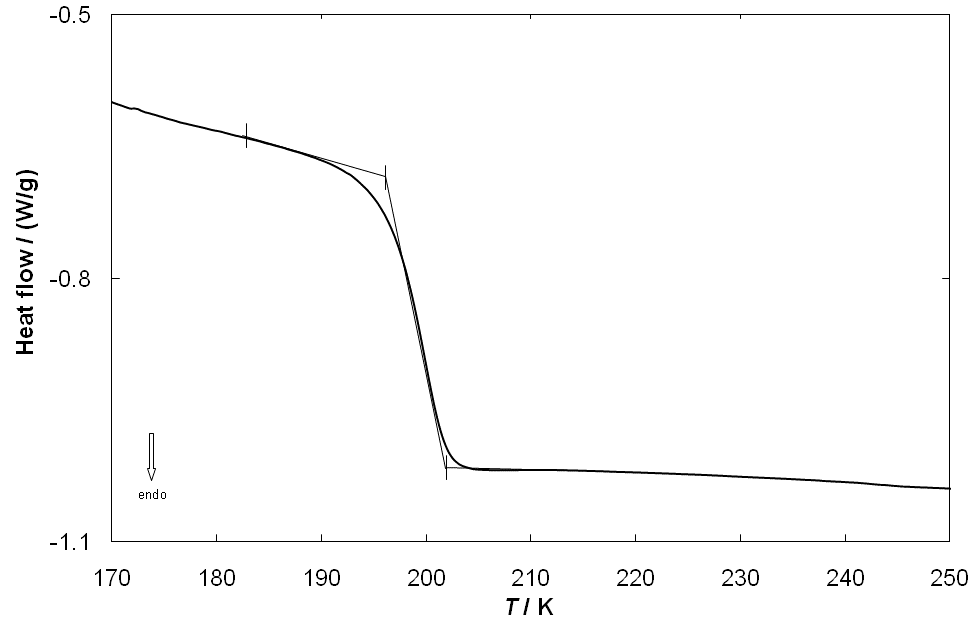


**Fig. 1S** TG/DTA analysis of [BMPy][SCN]; decomposition of IL


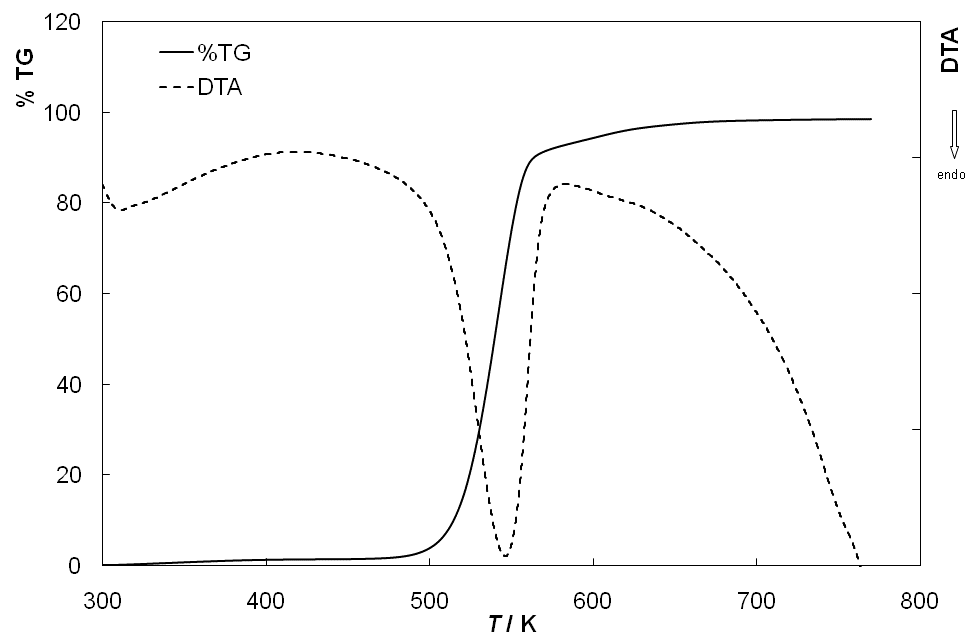


**Fig. 2S a** Density *ρ* for the {[BMIM][SCN] (1) + water (2)} binary systems as a function of temperature at different approximated mass fractions of IL : ●, 1.0000; ○, 0.9002;
▲, 0.7993; ∆, 0.6831; ■, 0.5654; □, 0.4704; *, 0.3574; ♦, 0.2799; ◊, 0.2051;
X, 0.1267; -, 0.0556;
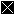
, 0.0000

**a**

**Fig. 2S b** Density, *ρ* for the {[BMPy][SCN] (1) + water (2)} binary systems as a function of temperature at different approximated mass fractions of IL : ●, 1.0000; ○, 0.8345; ▲, 0.6203; ∆, 0.4331; ■, 0.3001; □, 0.2377; *, 0.1667; ♦, 0.0972; ◊, 0.0430; X, 0.0286; -, 0.0000

**b**

**Fig. 2S c** Density *ρ* for the {[BMPYR][SCN] (1) + water (2)} binary systems as a function of temperature at different approximated mass fractions of IL : ●, 1.0000; ○, 0.4748;
▲, 0.3366; ∆, 0.2170; ■, 0.1377; □, 0.0343; *, 0.0000

**c**

**Fig. 2S d** Density *ρ* for the {[BMPIP][SCN] (1) + water (2)} binary systems as a function of temperature at different approximated mass fractions of IL : ●, 1.0000; ○, 0.6259;
▲, 0.4889; ∆, 0.3698; ■, 0.2828; □, 0.1752; *, 0.0649, ♦, 0.0000

**d**

**Fig. 3S a** Dynamic viscosity *η* for the {[BMIM][SCN] (1) + water (2)} binary systems as a function of temperature at different approximated molar fractions of the IL: ●, 1.0000; ○, 0.9002; ▲, 0.7993; ∆, 0.6831; ■, 0.5654; □, 0.4704; *, 0.3574; ♦, 0.2799; ◊, 0.2051;
X, 0.1267; -, 0.0556;
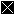
, 0.0000

**a**

**Fig. 3S b** Dynamic viscosity *η* for the {[BMPy][SCN] (1) + water (2)} binary systems as a function of temperature at different approximated molar fractions of the IL: ●, 1.0000; ○, 0.9436; ▲, 0.8345; ∆, 0.7220; ■, 0.6203; □, 0.5372; *, 0.4331; ♦, 0.3636; ◊, 0.2377; X, 0.1667; -, 0.0972;
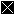
, 0.0000

**b**

**Fig. 3S c** Dynamic viscosity *η* for the {[BMPYR][SCN] (1) + water (2)} binary systems as a function of temperature at different approximated molar fractions of the IL: ●, 1.0000; ○, 0.9147; ▲, 0.7797; ∆, 0.6445; ■, 0.4748; □, 0.3366; *, 0.2170; ♦, 0.1377; ◊, 0.0000

**c**

**Fig. 3S d** Dynamic viscosity *η* for the {[BMPIP][SCN] (1) + water (2)} binary systems as a function of temperature at different approximated molar fractions of the IL: ●, 1.0000; ○, 0.7226; ▲, 0.6259; ∆, 0.4889; ■, 0.3698; □, 0.2828; *, 0.1752; ♦, 0.0000.

**d**

**Fig. 4S a** Density $\rho$for the {[BMPy][SCN] (1) + water (2)} system, as a function of mole fraction of the IL, at different temperatures: ●, 298.15 K; ○, 308.15 K; ▲, 318.15 K; ∆, 328.15 K; *, 338.15 K; ♦, 348.15 K

**a**

**Fig. 4S b** Density $\rho$for the {[BMPYR][SCN] (1) + water (2)} system, as a function of mole fraction of the IL, at different temperatures: ●, 298.15 K; ○, 308.15 K; ▲, 318.15 K; ∆, 328.15 K; *, 338.15 K; ♦, 348.15 K

**b**

**Fig. 4S c** Density $\rho$for the {[BMPIP][SCN] (1) + water (2)} system, as a function of mole fraction of the IL, at different temperatures: ▲, 318.15 K; ∆, 328.15 K; *, 338.15 K; ♦, 348.15 K

**c**

**Fig. 5S a** Dynamic viscosity $\eta$ for the {[BMPy][SCN] (1) + water (2)} binary system as a function of mole fraction of the IL, at a different temperatures: ●, 298.15 K; ○, 308.15 K; ▲, 318.15 K; ∆, 328.15 K; *, 338.15 K; ♦, 348.15

**a**

**Fig. 5S b** Dynamic viscosity $\eta$ for the {[BMPYR][SCN] (1) + water (2)} binary system as a function of mole fraction of the IL, at a different temperatures: ●, 298.15 K; ○, 308.15 K; ▲, 318.15 K; ∆, 328.15 K; *, 338.15 K; ♦, 348.15 K

**b**

**Fig. 5S c** Dynamic viscosity $\eta$ for the {[BMPIP][SCN] (1) + water (2)} binary system as a function of mole fraction of the IL, at a different temperatures: ▲, 318.15 K; ∆, 328.15 K; *, 338.15 K; ♦, 348.15 K

**c**

**Fig. 6S a** Viscosity deviation ∆*η* for the {[BMIM][SCN] (1) + water (2)} binary system at different temperatures: ●, 298.15 K; ○, 308.15 K; ▲, 318.15 K; ∆, 328.15 K; *, 338.15 K;
♦, 348.15 K. Solid lines represent the corresponding correlations by the Redlich – Kister equation

**a**

**Fig. 6S b** Viscosity deviation ∆*η* for the {[BMPYR][SCN] (1) + water (2)} binary system at different temperatures: ●, 298.15 K; ○, 308.15 K; ▲, 318.15 K; ∆, 328.15 K; *, 338.15 K; ♦, 348.15 K. Solid lines represent the corresponding correlations by the Redlich – Kister equation

**b**

**Fig. 6S c** Viscosity deviation ∆*η* for the {[BMPIP][SCN] (1) + water (2)} binary system at different temperatures: ▲, 318.15 K; ∆, 328.15 K; *, 338.15 K; ♦, 348.15 K. Solid lines represent the corresponding correlations by the Redlich – Kister equation

**c**

**Fig. 7S a** Plot of the isobaric expansivity *α* of the {[BMPy][SCN] (1) + water (2)} binary system against mole fraction *x*_1_ at different temperatures: (**1**) 298.15 K; (**2**) 308.15 K; (**3**) 318.15 K; (**4**) 328.15 K; (**5**) 338.15 K; (**6**) 348.15

6

5

4

3

2

1

**a**

**Fig. 7S b** Plot of the isobaric expansivity *α* of the {[BMPYR][SCN] (1) + water (2)} binary system against mole fraction *x*_1_ at different temperatures: (**1**) 298.15 K; (**2**) 308.15 K; (**3**) 318.15 K; (**4**) 328.15 K; (**5**) 338.15 K; (**6**) 348.15 K

6

5

4

3

2

1

**b**

**Fig. 7S c** Plot of the isobaric expansivity *α* of the {[BMPIP][SCN] (1) + water (2)} binary system against mole fraction *x*_1_ at different temperatures: (**1**) 318.15 K; (**2**) 328.15 K; (**3**) 338.15 K; (**4**) 348.15 K

4

3

2

1

**c**

**Fig. 8S a** Plot of the excess isobaric expansivity *α*^E^ of the {[BMPy][SCN] (1) + water (2)} binary system against mole fraction *x*_1_ at different temperatures: (**1**) 298.15 K; (**2**) 308.15 K; (**3**) 318.15 K; (**4**) 328.15 K; (**5**) 338.15 K; (**6**) 348.15 K

1

2

3

4

5

6

**a**

**Fig. 8S b** Plot of the excess isobaric expansivity *α*^E^ of the {[BMPYR][SCN] (1) + water (2)} binary system against mole fraction *x*_1_ at different temperatures: (**1**) 298.15 K; (**2**) 308.15 K;
(**3**) 318.15 K; (**4**) 328.15 K; (**5**) 338.15 K; (**6**) 348.15 K

1

2

3

4

5

6

**b**

**Fig. 8S c** Plot of the excess isobaric expansivity *α*^E^ of the {[BMPIP][SCN] (1) + water (2)} binary system against mole fraction *x*_1_ at different temperatures: (**1**) 318.15 K; (**2**) 328.15 K;
(**3**) 338.15 K; (4) 348.15 K

1

2

3

4

**c**
